# Synthesis of tetrahydro-β-carbolines from 2-indolylmethyl azides and propargylic alcohols†

**DOI:** 10.1039/d1ra03022a

**Published:** 2021-06-01

**Authors:** Haiting Yin, Qin Ma, Yushan Wang, Xiaoxia Gu, Zhijun Feng, Yunjun Wu, Ming Wang, Shaoyin Wang

**Affiliations:** Department of Chemistry, Institute of Synthesis and Application of Medical Materials, Chunhui Scientific Research Interest Group, Wannan Medical College Wuhu Anhui 241002 China wsychem@163.com

## Abstract

A facile and efficient route to tetrahydro-β-carbolines from 2-indolylmethyl azides and propargylic alcohols *via* acid-catalyzed dehydrative annulation reactions is described. This reaction proceeds through a cascade sequence of Friedel–Crafts-type alkylation followed by intramolecular “Click” reaction, involving the formation of multiple chemical bonds in a single operation with excellent atom-economy and broad functional group tolerance.

## Abstract 2

The synthesis of tetrahydro-β-carbolines from 2-indolylmethyl azides and propargylic alcohols *via* acid-catalyzed dehydrative [4 + 2]-annulation reaction is described.

## Graphical abstract

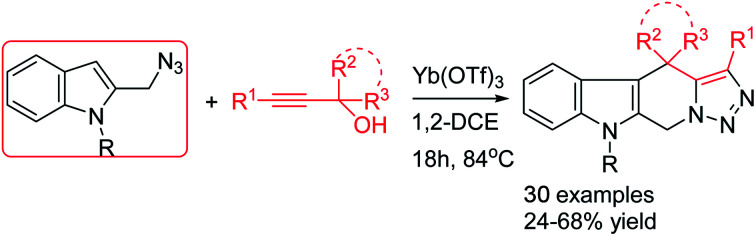

## Introduction

Tetrahydro-β-carboline (THBC) derivatives are very important alkaloids with various biological activities and pharmaceutical applications and with extensive occurrence in natural products.^1^ Consequently, intense efforts have been devoted to the development of efficient methods for their synthesis and significant progress has been achieved. However, most current methods such as the classical Pictet–Spengler reaction are limited in product scope as mostly only products with substitution at the 1-position of the THBC ring are accessible.^2^ Therefore, the development of methods with wide product diversity is still highly desirable.

**Scheme 1:**
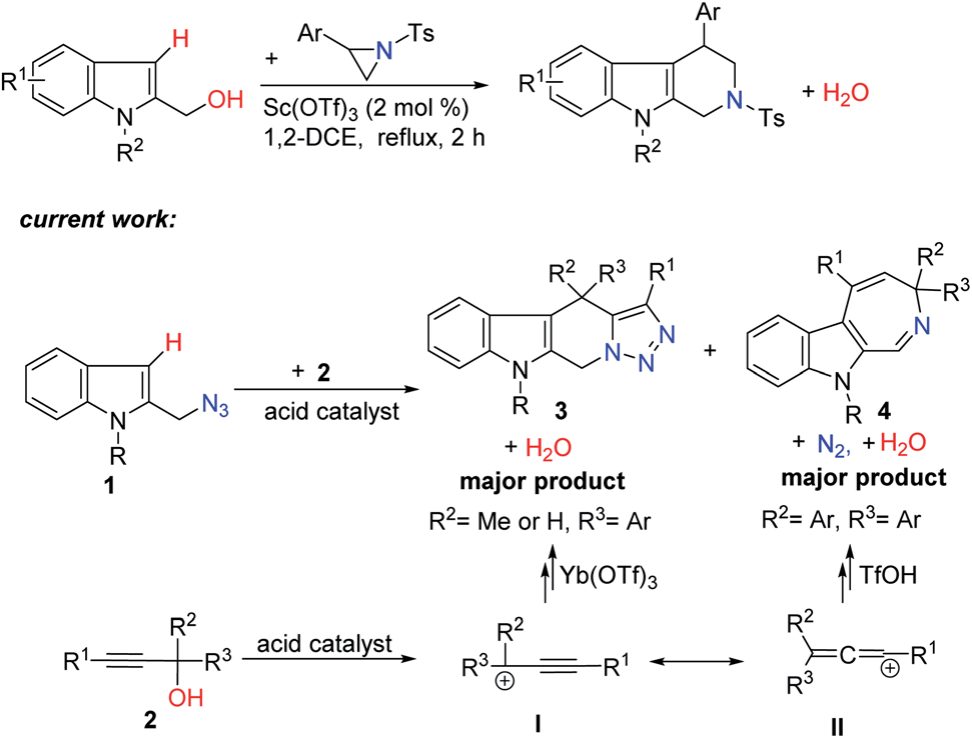
Lewis acid-catalyzed dehydrative annulation way to tetrahydro-β-carboline derivatives.

Recently, our group have developed cascade processes using *N*-sulfonyl aziridines as a nitrogen source and simple electron-rich benzylic alcohols as a versatile three-carbon synthon for the synthesis of tetrahydro-β-carbolines and tetrahydroisoquinolines.^3^ We then reasoned that the use of 2-indolylmethyl azides as a nitrogen source and the use of readily available propargylic alcohols as a three-carbon synthon may enable access to compounds with novel structures not easily accessible by other methods. One major challenge associated with this strategy is the competitive Friedel–Crafts-type alkylations of 1 between the two resonance forms I and II of the carbocation intermediate generated from 2 in the presence of an acid catalyst (Scheme 1).^4^ We disclose here that the selectivity of this process hinges on both the use of different acid catalysts and the substituents on propargylic alcohols, providing a new atom-economic way to tetracyclic tetrahydro-β-carboline derivatives with a fused triazole structure and indole azepines.^5^

## Results and discussion

**Fig. 1:**
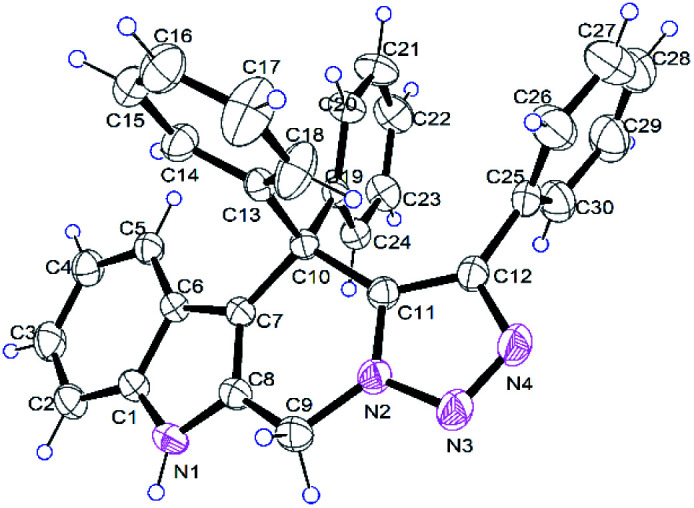
Crystal structure of compound 3aa.

The reaction of 2-(azidomethyl)-1*H*-indole 1a and propargylic alcohol 2a was selected as a model reaction for optimization of reaction conditions (Table 1). Using 1,2-dichloroethane (1,2-DCE) as solvent, four different rare-earth metal triflates were screened, and two products 3aa and 4aa were generally obtained (Table 1, entries 2–5). Yb(OTf)_3_ was found to be the most efficient catalyst for this reaction, providing the highest overall yield of tetrahydro-β-carboline 3 and azepine 4 (Table 1, entry 5). No reaction occurred in the absence of the catalyst or when the reaction was performed at room temperature (Table 1, entries 1 and 6). Changing the solvent to toluene, DMF, 1,4-dioxane, or THF gave inferior results (Table 1, entries 7–10). Further screen of catalyst loading amount revealed that 10 mol% was optimal for the reaction, while lower (5 mol%) or higher (20 mol%) loadings all led to no improvement in both overall yield and product selectivity (Table 1, entries 11 and 12). Interestingly, when the Brønsted acid TfOH was used as the catalyst, the azepine 4aa was the major product, albeit in a relatively lower overall yield (Table 1, entry 13). It is worth mentioning that the reaction is tolerant of moisture and air and could be performed in commercial solvents under open air. The structures of the products 3aa (Fig. 1) and 4aa were additionally confirmed by X-ray crystallographic analysis (see ESI† for details).

**Table 1:** Screening of the reaction conditions^a^

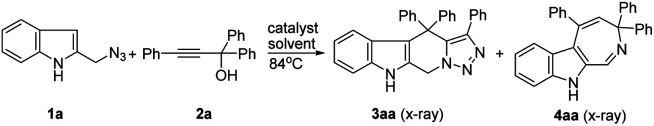					
Entry	Catalyst (mol%)	Solvent	Time (h)	Yield^b^ (%)	
				3aa	4aa
1	No catalyst	1,2-DCE	24	0	0
2	Sc(OTf)_3_ (10%)	1,2-DCE	14	20	45
3	Y(OTf)_3_ (10%)	1,2-DCE	14	19	42
4	La(OTf)_3_ (10%)	1,2-DCE	24	15	35
5	Yb(OTf)_3_ (10%)	1,2-DCE	18	25	51
6^c^	Yb(OTf)_3_ (10%)	1,2-DCE	24	0	0
7^d^	Yb(OTf)_3_ (10%)	Toluene	18	15	46
8^d^	Yb(OTf)_3_ (10%)	DMF	24	0	0
9^d^	Yb(OTf)_3_ (10%)	1,4-Dioxane	18	16	41
10^e^	Yb(OTf)_3_ (10%)	THF	18	22	49
11	Yb(OTf)_3_ (5%)	1,2-DCE	18	20	36
12	Yb(OTf)_3_ (20%)	1,2-DCE	18	23	50
13	TfOH (20%)	1,2-DCE	18	Trace	53
14	TsOH (20%)	1,2-DCE	18	15	35
15^f^	Yb(OTf)_3_ (10%)	1,2-DCE	18	25	50

a Reaction conditions: 1a (0.5 mmol), 2a (0.5 mmol), solvent (5 mL), the reaction was monitored by TLC.

b Yield of the isolated product.

c Reaction was run at 25 °C.

d Reaction was run at 90 °C.

e Reaction was run at 66 °C.

f Dried 1,2-DCE under argon atmosphere.

**Scheme 2:**
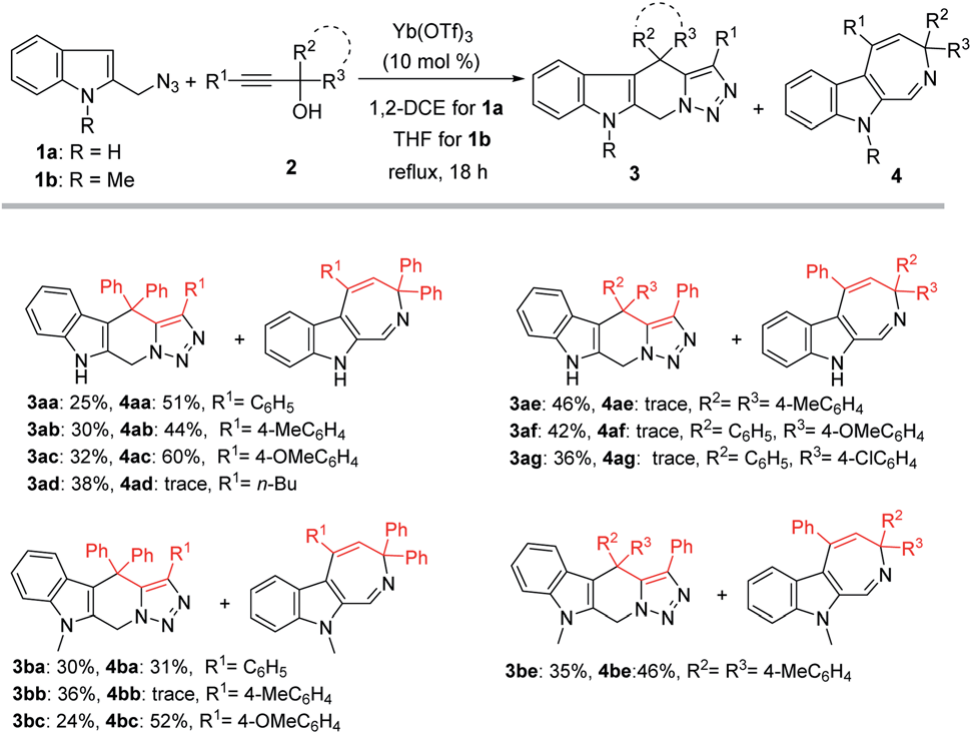
Scope study with different propargylic alcohols 2. ^*a*^Reaction conditions: 1a (0.5 mmol), 2 (0.5 mmol), Yb(OTf)_3_ (0.05 mmol), solvent (5 mL), reflux. ^*b*^Isolated yield refers to azide.

With the optimized reaction conditions in hand, a series of propargylic alcohols 2 were reacted with 2-(azidomethyl)-1*H*-indole 1a to examine the reaction scope with regard to the formation of tetrahydro-β-carboline 3 and azepine 4. In general, the two products were produced in poor ratios, with the yields of 3 ranging from 25% to 46%. Substrate 2d derived from an aliphatic alkyne (R^1^ = *n*-Bu) was also suitable in this reaction (Scheme 2, 3ad), while only trace of the corresponding product 4ad was detected. As for the products azepines 4, propargylic alcohols 2 bearing electron-donating substituents on the aryl group (R^1^) provided higher yields than others (Scheme 2, 4ac–4ad).

Next, *N*-methyl-2-(azidomethyl)-1*H*-indole 1b was also examined in the reaction, under similar reaction conditions except that the reactions were performed at a lower reaction temperature using THF as solvent. In general, comparable results were obtained as to those obtained with 1a.

**Scheme 3:**
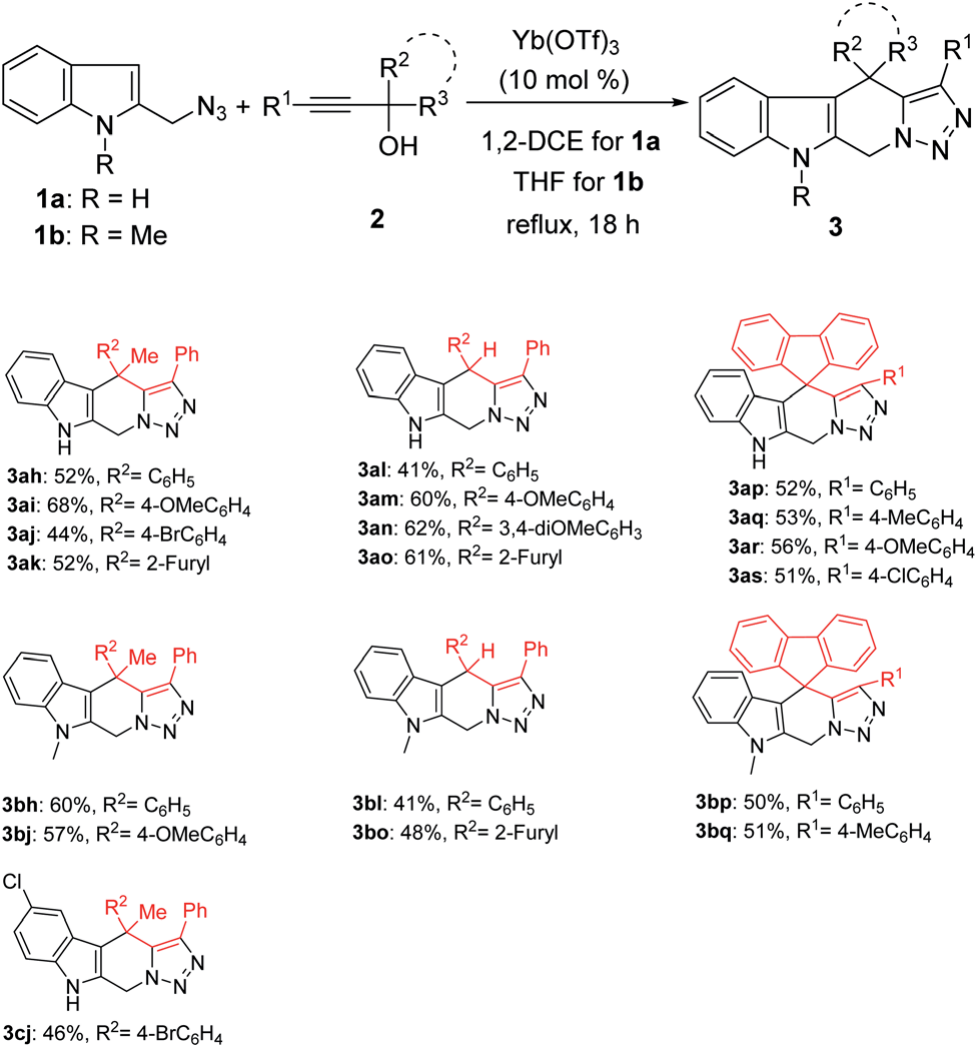
Scope study with other propargylic alcohols. ^*a*^Reaction conditions: 1 (0.5 mmol), 2 (0.5 mmol), Yb(OTf)_3_ (0.05 mmol), solvent (5 mL), reflux. ^*b*^Isolated yield refers to azide.

Notably, when tertiary propargylic alcohols 2 with either of the two substituents (R^2^, R^3^) being an alkyl group (Me) or secondary propargylic alcohols were used in this reaction, only tetrahydro-β-carbolines 3 were isolated in moderate yields (Scheme 3). The reduced steric hindrance in the carbocation intermediate I (Scheme 1) might be partly responsible for this product selectivity. Propargylic alcohols 2 bearing electron-donating substituents on aryl groups (R^2^) provided higher yields than those with electron-withdrawing ones (Scheme 3, 3ah–3ao). Moreover, when 9-fluorenyl-substituted propargylic alcohols 2p–2s were subjected to the reaction conditions, spirocyclic products 3ap–3as could be formed in the yields of 51–56% as the only products.

**Scheme 4:**
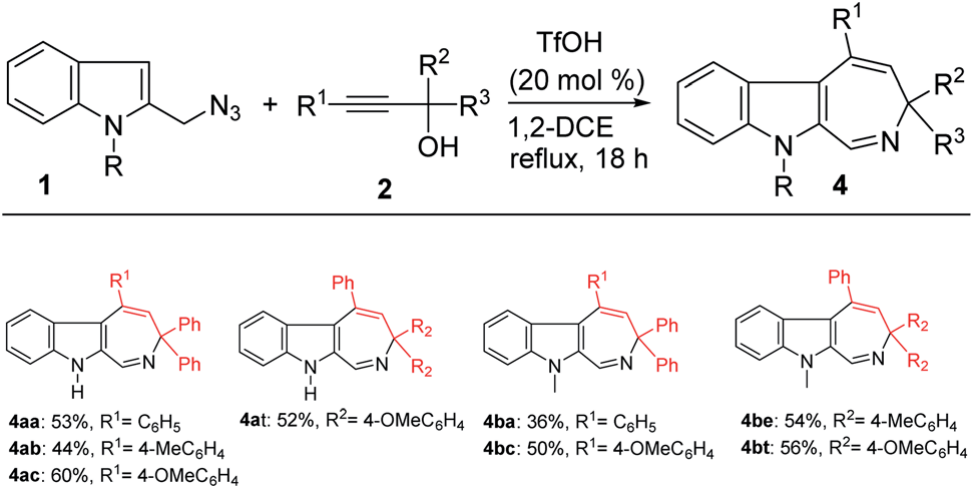
Scope study on the formation of the indole azepines. ^*a*^Reaction conditions: 1 (0.5 mmol), 2 (0.5 mmol), TfOH (0.1 mmol), 1,2-DCE (5 mL), reflux. ^*b*^Isolated yield.

We also probed the reaction scope with regard to the synthesis of indole azepines *via* the TfOH-catalyzed formal [4 + 3]-annulation route. In general, only propargylic alcohols 2 bearing an electron-donating substituent on one of the aryl groups (R^1^, R^2^, R^3^) worked in this system to provide the corresponding indole azepines in moderate yields (Scheme 4). While the use of 1a or 1b also gave comparable results, the corresponding tetrahydro-β-carboline products 3 were detected in only trace amount in these cases.

**Scheme 5:**
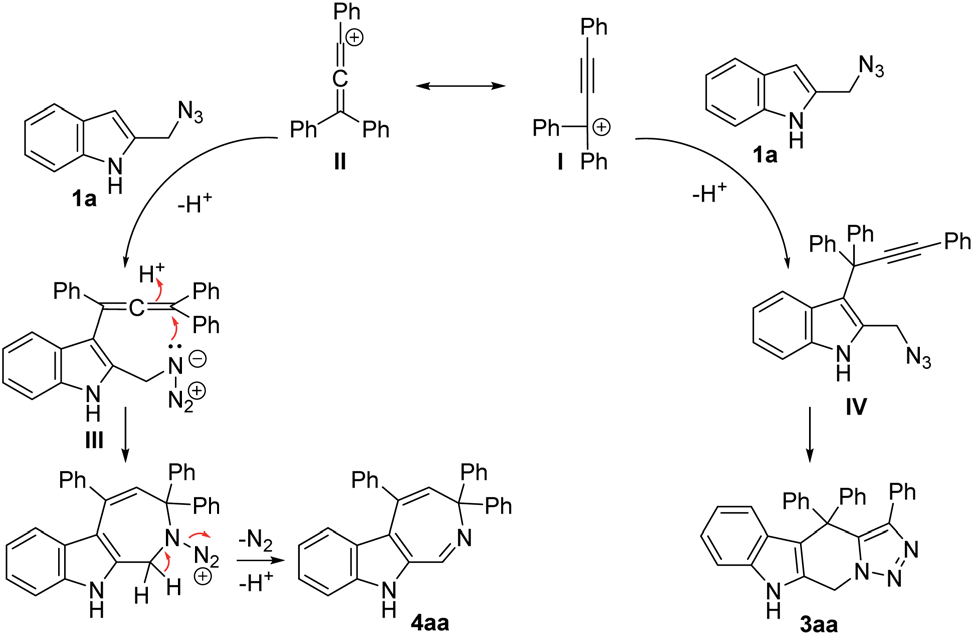
A possible mechanism for the dehydrative annulations.

Based on the above experimental results, a plausible mechanism for the present cascade reactions was proposed (Scheme 5). First, in the presence of an acid catalyst, propargylic alcohol 2a would be converted to the propargylic carbocation I, which is in equilibrium with the allenic form II. Subsequent Friedel–Crafts-type reaction with 1a would form the propargylic intermediate IV (from I) or allenic intermediate III (from II). Then intermediate IV would be transformed to the final product tetrahydro-β-carboline 3aa by an intramolecular click reaction, while the allenic intermediate III would be transformed to the azepine product 4aa*via* an acid-catalyzed allene hydroamination with the concurrent release of a molecule of N_2_. Both the nature of the acid catalyst and the electronic and steric effects of the substituents on the propargylic alcohols have influence on the product selectivity of the reaction.

## Conclusions

In summary, two cascade reactions of 2-indolylmethyl azides and propargylic alcohols were developed by using simple Lewis acid and Brønsted acid catalysts. The good atom- and step-economy, readily available starting materials, easy operation, and mild reaction conditions render this method a useful alternative way to tetrahydro-β-carbolines and indole azepines. Efforts towards the utilization of the propargylic alcohols to the synthesis of other useful cyclic compounds are underway in our laboratories.

## Experimental section

### General comments

Infrared spectra were obtained on a FTIR spectrometer. ^1^H NMR spectra were recorded on 300 MHz or 400 MHz spectrometer in DMSO-*d*_6_ or CDCl_3_ solution and the chemical shifts were reported relative to internal standard TMS (0 ppm). The following abbreviations are used to describe peak patterns where appropriate: br = broad, s = singlet, d = doublet, t = triplet, q = quartet, m = multiplet. Coupling constants are reported in hertz (Hz). ^13^C NMR were recorded on 75 MHz or 100 MHz and referenced to the internal solvent signals (central peak is 40.00 ppm in DMSO-*d*_6_ or 77.00 ppm in CDCl_3_). HRMS data were obtained using ESI ionization. Melting points were measured with micro melting point apparatus.

The 2-(azidomethyl)-1*H*-indoles 1 were prepared from indole-2-methanols,^6^ and the indole-2-methanols were prepared from indole-2-carboxylates.^7^ The propargylic alcohols 2 were prepared from phenylacetylene and benzophenone according to the published methods.^8^ All commercially available reagents and solvents were used without further purification unless noted otherwise.

### General procedure for the synthesis of 3

A solution of indole 1 (0.5 mmol), propargylic alcohols 2 (0.5 mmol) and Yb(OTf)_3_ (0.05 mmol) in 1,2-DCE (5 mL, for 1a) at 84 °C or THF (5 mL, for 1b) at 66 °C was stirred under open air for 18 h. After being cooled down to room temperature, the mixture was diluted with ethyl acetate (50 mL), washed with saturated NaCl solution (10 mL) and dried over anhydrous Na_2_SO_4_. The solvent was evaporated and the crude product was purified by silica gel column chromatography with petroleum ether/ethyl acetate (1 : 1, v/v).

### General procedure for the synthesis of 4

A solution of 2-(azidomethyl)-1*H*-indole 1 (0.5 mmol), the propargylic alcohols 2 (0.5 mmol) and TfOH (0.05 mmol) in 1,2-DCE (5 mL) was stirred under air at 84 °C for 18 h. After being cooled down to room temperature, the mixture was diluted with ethyl acetate (50 mL), washed with saturated NaCl solution (10 mL) and dried over anhydrous Na_2_SO_4_. The solvent was evaporated and the crude product was purified by silica gel column chromatography with petroleum ether/ethyl acetate (6 : 1, v/v).

### Characterization data of products

#### 3,4,4-Triphenyl-9,10-dihydro-4*H*-[1,2,3]triazolo[1′,5′:1,6]pyrido[3,4-*b*]indole (3aa)

White solid (55 mg, 25%); mp 349–350 °C; ^1^H NMR (300 MHz, DMSO-*d*_6_) *δ* 11.51 (s, 1H), 7.40 (d, *J* = 8.1 Hz, 1H), 7.26–7.10 (m, 7H), 7.10–6.96 (m, 7H), 6.87–6.70 (m, 3H), 6.51 (d, *J* = 8.0 Hz, 1H), 5.89 (s, 2H); ^13^C NMR (75 MHz, DMSO-*d*_6_) *δ* 144.3, 143.2, 137.8, 137.6, 132.2, 129.6, 129.1, 129.0, 128.4, 127.9, 127.8, 127.4, 125.0, 121.8, 119.2, 119.8, 115.0, 112.3, 51.4, 45.2; IR (KBr) *ν* 3274, 3055, 1618, 1487, 1328, 1130, 743, 700 cm^−1^; HRMS: *m*/*z* calcd for ([C_30_H_22_N_4_ + H]^+^): 439.1917; found: 439.1914.

#### 4,4-Diphenyl-3-(*p*-tolyl)-9,10-dihydro-4*H*-[1,2,3]triazolo[1′,5′:1,6]pyrido[3,4-*b*]indole (3ab)

White solid (68 mg, 30%); mp 275–276 °C; ^1^H NMR (300 MHz, DMSO-*d*_6_) *δ* 11.48 (s, 1H), 7.46–7.32 (m, 1H), 7.22–7.07 (m, 6H), 7.07–6.95 (m, 5H), 6.83 (d, *J* = 7.9 Hz, 2H), 6.72 (dd, *J* = 8.0, 7.2 Hz, 1H), 6.62 (d, *J* = 8.0 Hz, 2H), 6.49 (d, *J* = 8.0 Hz, 1H), 5.86 (s, 2H), 2.18 (s, 3H); ^13^C NMR (75 MHz, DMSO-*d*_6_) *δ* 144.3, 143.4, 137.9, 137.7, 137.2, 129.5, 129.4, 129.2, 129.0, 128.5, 128.5, 127.5, 125.1, 121.9, 120.0, 119.8, 115.0, 112.4, 51.4, 45.2, 21.3; IR (KBr) *ν* 3047, 2922, 1636, 1491, 1342, 1021, 743, 700 cm^−1^; HRMS: *m*/*z* calcd for ([C_31_H_24_N_4_ + H]^+^): 453.2074; found: 453.2071.

#### 3-(4-Methoxyphenyl)-4,4-diphenyl-9,10-dihydro-4*H*-[1,2,3]triazolo[1′,5′:1,6]pyrido[3,4-*b*]indole (3ac)

White solid (75 mg, 32%); mp 310–311 °C; ^1^H NMR (300 MHz, DMSO-*d*_6_) *δ* 11.47 (s, 1H), 7.37 (d, *J* = 8.1 Hz, 1H), 7.21–7.08 (m, 6H), 7.07–6.95 (m, 5H), 6.73 (t, *J* = 7.6 Hz, 1H), 6.68–6.62 (m, 2H), 6.61–6.54 (m, 2H), 6.50 (d, *J* = 8.0 Hz, 1H), 5.85 (s, 2H), 3.65 (s, 3H); ^13^C NMR (75 MHz, DMSO-*d*_6_) *δ* 144.3, 143.4, 137.9, 137.7, 137.2, 129.5, 129.4, 129.2, 129.0, 128.5, 128.5, 127.5, 125.1, 121.9, 120.0, 119.8, 115.0, 112.4, 51.4, 45.2, 21.3; IR (KBr) *ν* 3273, 3055, 2924, 1618, 1498, 1249, 1173, 1025, 739, 703 cm^−1^; HRMS: *m*/*z* calcd for ([C_31_H_24_N_4_O + H]^+^): 469.2032; found: 469.2028.

#### 3-Butyl-4,4-diphenyl-9,10-dihydro-4*H*-[1,2,3]triazolo[1′,5′:1,6]pyrido[3,4-*b*]indole (3ad)

White solid (80 mg, 38%); mp 257–258 °C; ^1^H NMR (300 MHz, DMSO-*d*_6_) *δ* 11.49 (s, 1H), 7.41 (d, *J* = 8.1 Hz, 1H), 7.34–7.23 (m, 6H), 7.15–6.98 (m, 5H), 6.78 (t, *J* = 7.6 Hz, 1H), 6.51 (d, *J* = 8.0 Hz, 1H), 5.72 (s, 2H), 1.94 (t, *J* = 7.2 Hz, 2H), 1.21–1.07 (m, 2H), 1.07–0.92 (m, 2H), 0.64 (t, *J* = 7.2 Hz, 3H); ^13^C NMR (75 MHz, DMSO-*d*_6_) *δ* 143.9, 137.5, 136.7, 129.9, 128.9, 128.8, 127.6, 125.5, 121.8, 119.8, 114.9, 112.3, 50.9, 44.8, 30.7, 25.5, 22.4, 14.0; IR (KBr) *ν* 3049, 2922, 1618, 1508, 1460, 1108, 743, 624 cm^−1^; HRMS: *m*/*z* calcd for ([C_28_H_26_N_4_ + H]^+^): 419.2230; found: 419.2232.

#### 3-Phenyl-4,4-di-*p*-tolyl-9,10-dihydro-4*H*-[1,2,3]triazolo[1′,5′:1,6]pyrido[3,4-*b*]indole (3ae)

White solid (107 mg, 46%); mp 358–359 °C; ^1^H NMR (300 MHz, DMSO-*d*_6_) *δ* 11.45 (s, 1H), 7.38 (d, *J* = 8.1 Hz, 1H), 7.16 (t, *J* = 7.3 Hz, 1H), 7.11–6.97 (m, 3H), 6.97–6.83 (m, 8H), 6.81–6.69 (m, 3H), 6.55 (d, *J* = 8.0 Hz, 1H), 5.85 (s, 2H), 2.19 (s, 6H); ^13^C NMR (75 MHz, DMSO-*d*_6_) *δ* 144.2, 140.5, 138.2, 137.6, 136.4, 132.3, 129.6, 129.0, 128.9, 128.8, 127.7, 125.0, 121.8, 120.1, 119.7, 115.2, 112.2, 50.8, 45.1, 21.0; IR (KBr) *ν* 3049, 2922, 1636, 1509, 1133, 1007, 747, 620 cm^−1^; HRMS: *m*/*z* calcd for ([C_32_H_26_N_4_ + H]^+^): 467.2230; found: 467.2228.

#### 4-(4-Methoxyphenyl)-3,4-diphenyl-9,10-dihydro-4*H*-[1,2,3]triazolo[1′,5′:1,6]pyrido[3,4-*b*]indole (3af)

White solid (98 mg, 42%); mp 320–321 °C; ^1^H NMR (300 MHz, DMSO-*d*_6_) *δ* 11.49 (s, 1H), 7.40 (d, *J* = 8.1 Hz, 1H), 7.28–6.98 (m, 9H), 6.95 (d, *J* = 8.8 Hz, 2H), 6.89–6.73 (m, 3H), 6.69 (d, *J* = 8.8 Hz, 2H), 6.55 (d, *J* = 8.0 Hz, 1H), 5.88 (s, 2H), 3.65 (s, 3H); ^13^C NMR (75 MHz, DMSO-*d*_6_) *δ* 144.2, 140.5, 138.2, 137.6, 136.4, 132.3, 129.6, 129.0, 128.9, 128.8, 127.7, 125.0, 121.8, 120.1, 119.7, 115.2, 112.2, 50.8, 45.1, 21.0. IR (KBr) *ν* 2991, 2887, 1636, 1509, 1249, 1180, 750, 696 cm^−1^; HRMS: *m*/*z* calcd for ([C_31_H_24_N_4_ + H]^+^): 469.2023; found: 469.2024.

#### 4-(4-Chlorophenyl)-3,4-diphenyl-9,10-dihydro-4*H*-[1,2,3]triazolo[1′,5′:1,6]pyrido[3,4-*b*]indole (3ag)

White solid (85 mg, 36%); mp 315–316 °C; ^1^H NMR (300 MHz, DMSO-*d*_6_) *δ* 11.54 (s, 1H), 7.40 (d, *J* = 8.1 Hz, 1H), 7.25–7.12 (m, 6H), 7.12–6.94 (m, 7H), 6.87–6.72 (m, 3H), 6.54 (d, *J* = 8.0 Hz, 1H), 5.89 (AB, *J* = 18.9 Hz, 2H); ^13^C NMR (75 MHz, DMSO-*d*_6_) *δ* 144.2, 140.5, 138.2, 137.6, 136.4, 132.3, 129.6, 129.0, 128.9, 128.8, 127.7, 125.0, 121.8, 120.1, 119.7, 115.2, 112.2, 50.8, 45.1, 21.0. IR (KBr) *ν* 3028, 2908, 1598, 1483, 1342, 1163, 661, 547 cm^−1^; HRMS: *m*/*z* calcd for ([C_30_H_21_ClN_4_ + H]^+^): 473.1528; found: 473.1525.

#### 4-Methyl-3,4-diphenyl-9,10-dihydro-4*H*-[1,2,3]triazolo[1′,5′:1,6]pyrido[3,4-*b*]indole (3ah)

White solid (98 mg, 52%); mp 310–311 °C; ^1^H NMR (300 MHz, DMSO-*d*_6_) *δ* 11.34 (s, 1H), 7.37 (d, *J* = 8.1 Hz, 1H), 7.34–7.12 (m, 9H), 7.03 (t, *J* = 7.5 Hz, 1H), 6.90–6.74 (m, 3H), 6.03 (AB *J* = 16.9 Hz, 2H), 1.92 (s, 3H). ^13^C NMR (75 MHz, DMSO-*d*_6_) *δ* 146.9, 142.6, 139.1, 137.9, 132.6, 129.3, 128.7, 128.4, 127.6, 127.0, 125.9, 124.1, 121.9, 119.4, 119.3, 114.0, 112.1, 45.0, 24.8. IR (KBr) *ν* 2987, 1625, 1132, 1242, 1007, 746, 703 cm^−1^; HRMS: *m*/*z* calcd for ([C_25_H_20_N_4_ + H]^+^): 377.1761; found: 377.1757.

#### 4-(4-Methoxyphenyl)-4-methyl-3-phenyl-9,10-dihydro-4*H*-[1,2,3]triazolo[1′,5′:1,6]pyrido[3,4-*b*]indole (3ai)

White solid (138 mg, 68%); mp 315–316 °C; ^1^H NMR (300 MHz, DMSO-*d*_6_) *δ* 11.31 (s, 1H), 7.37 (d, *J* = 8.0 Hz, 1H), 7.33–7.15 (m, 6H), 7.03 (t, *J* = 7.5 Hz, 1H), 6.89–6.73 (m, 5H), 6.01 (AB, *J* = 16.9 Hz, 2H), 3.70 (s, 3H), 1.90 (s, 3H). ^13^C NMR (75 MHz, DMSO) *δ* 158.1, 142.6, 139.3, 138.9, 137.9, 132.7, 129.3, 128.7, 128.3, 128.3, 125.8, 124.2, 121.9, 119.5, 119.3, 114.3, 113.9, 112.0, 55.5, 45.0, 40.8, 25.1. IR (KBr) *ν* 2948, 2832, 1607, 1509, 1260, 1184,1021, 837, 739, 703 cm^−1^; HRMS: *m*/*z* calcd for ([C_26_H_22_N_4_O + H]^+^): 407.1866; found: 407. 1867.

#### 4-(4-Bromophenyl)-4-methyl-3-phenyl-9,10-dihydro-4*H*-[1,2,3]triazolo[1′,5′:1,6]pyrido[3,4-*b*]indole (3aj)

White solid (100 mg, 44%); mp 327–328 °C, ^1^H NMR (300 MHz, DMSO-*d*_6_) *δ* 11.39 (s, 1H), 7.47–7.35 (m, 3H), 7.35–7.12 (m, 6H), 7.05 (t, *J* = 7.6 Hz, 1H), 6.92–6.78 (m, 3H), 6.03 (AB, 16.9 Hz, 2H), 1.91 (s, 3H). ^13^C NMR (75 MHz, DMSO-*d*_6_) *δ* 146.1, 142.7, 138.5, 137.9, 132.4, 131.4, 130.0, 129.4, 128.4, 126.2, 123.9, 122.0, 120.1, 119.4, 119.2, 113.5, 112.1, 45.0, 25.1. IR (KBr) *ν* 2942, 1621, 1487, 1328, 1075, 1010, 743, 700 cm^−1^; HRMS: *m*/*z* calcd for ([C_25_H_19_BrN_4_ + H]^+^): 455.0866; found: 455.0865.

#### 4-(Furan-2-yl)-4-methyl-3-phenyl-9,10-dihydro-4*H*-[1,2,3]triazolo[1′,5′:1,6]pyrido[3,4-*b*]indole (3ak)

White solid (95 mg, 52%); mp 290–291 °C; ^1^H NMR (300 MHz, DMSO-*d*_6_) *δ* 11.37 (s, 1H), 7.44–7.34 (m, 3H), 7.34–7.24 (m, 3H), 7.11–7.02 (m, 1H), 7.00–6.84 (m, 3H), 6.52–6.45 (m, 1H), 6.40–6.35 (m, 1H), 5.92 (AB, *J* = 17.1 Hz, 2H), 1.86 (s, 3H). ^13^C NMR (75 MHz, DMSO) *δ* 157.7, 143.2, 142.5, 137.8, 135.9, 132.4, 129.2, 128.6, 128.5, 126.6, 124.2, 122.1, 119.6, 119.3, 112.2, 111.1, 110.8, 106.9, 45.0, 37.5, 25.3. IR (KBr) *ν* 2980, 2893, 1625, 1459, 1336, 1234, 1007, 743, 700 cm^−1^; HRMS: *m*/*z* calcd for ([C_23_H_18_N_4_O + H]^+^): 367.1553; found: 367.1558.

#### 3,4-Diphenyl-9,10-dihydro-4*H*-[1,2,3]triazolo[1′,5′:1,6]pyrido[3,4-*b*]indole (3al)

White solid (74 mg, 41%); mp 330–331 °C; 1H NMR (300 MHz, DMSO-*d*_6_) *δ* 11.35 (s, 1H), 7.74 (d, *J* = 7.2 Hz, 2H), 7.48 (d, *J* = 7.8 Hz, 1H), 7.43–7.20 (m, 6H), 7.20–6.97 (m, 4H), 6.92 (t, *J* = 7.5 Hz, 1H), 6.24–6.02 (m, 2H), 6.00–5.81 (m, 1H), ^13^C NMR (75 MHz, DMSO-*d*_6_) *δ* 143.0, 141.9, 137.6, 133.7, 131.5, 128.8, 128.7, 128.5, 128.0, 127.3, 127.0, 125.2, 122.1, 119.4, 119.1, 111.9, 109.2, 45.3, 37.2. IR (KBr) *ν* 2933, 1607, 1516, 1462, 1271, 1133, 1021, 739, 700 cm^−1^; HRMS: *m*/*z* calcd for ([C_24_H_18_N_4_ + H]^+^): 363.1604; found: 363.1604.

#### 4-(4-Methoxyphenyl)-3-phenyl-9,10-dihydro-4*H*-[1,2,3]triazolo[1′,5′:1,6]pyrido[3,4-*b*]indole (3am)

White solid (118 mg, 60%); mp 282–283 °C; ^1^H NMR (300 MHz, DMSO-*d*_6_) *δ* 11.31 (s, 1H), 7.82–7.72 (m, 2H), 7.47 (d, *J* = 7.8 Hz, 1H), 7.41–7.30 (m, 3H), 7.30–7.14 (m, 3H), 7.06 (t, *J* = 7.2 Hz, 1H), 6.92 (t, *J* = 7.2 Hz, 1H), 6.65 (d, *J* = 8.7 Hz, 2H), 6.17–6.01 (m, 2H), 5.94–5.83 (m, 1H), 3.57 (s, 3H). ^13^C NMR (75 MHz, DMSO-*d*_6_) *δ* 158.2, 141.8, 137.6, 135.0, 134.0, 131.6, 129.6, 128.8, 128.0, 127.3, 126.9, 125.3, 122.1, 119.4, 119.2, 114.1, 111.9, 109.6, 55.3, 45.3, 36.4. IR (KBr) *ν* 2930, 1610, 1513, 1459, 1260, 1032, 743, 700 cm^−1^; HRMS: *m*/*z* calcd for ([C_25_H_20_N_4_O + H]^+^): 393.1710; found: 393.1710.

#### 4-(3,4-Dimethoxyphenyl)-3-phenyl-9,10-dihydro-4*H*-[1,2,3]triazolo[1′,5′:1,6]pyrido[3,4-*b*]indole (3an)

White solid (131 mg, 62%); mp 301–302 °C; ^1^H NMR (300 MHz, DMSO-*d*_6_) *δ* 11.33 (s, 1H), 7.92–7.68 (m, 2H), 7.59–7.19 (m, 5H), 7.18–6.79 (m, 3H), 6.77–6.47 (m, 2H), 6.21–5.77 (m, 3H), 3.60 (s, 3H), 3.55 (s, 3H). ^13^C NMR (75 MHz, DMSO-*d*_6_) *δ* 148.5, 147.7, 142.0, 137.7, 135.3, 133.9, 131.7, 128.84, 128.0, 127.5, 127.1, 125.4, 122.1, 120.4, 119.4, 119.3, 112.9, 112.1, 111.9, 109.3, 56.0, 55.7, 45.3, 36.7. IR (KBr) *ν* 2933, 1607, 1516, 1462, 1271, 1133, 1021, 739, 700 cm^−1^; HRMS: *m*/*z* calcd for ([C_26_H_22_N_4_O_2_ + H]^+^): 423.1816; found: 423.1815.

#### 4-(Furan-2-yl)-3-phenyl-9,10-dihydro-4*H*-[1,2,3]triazolo[1′,5′:1,6]pyrido[3,4-*b*]indole (3ao)

White solid (107 mg, 61%); mp 323–324 °C; ^1^H NMR (300 MHz, DMSO-*d*_6_) *δ* 11.40 (s, 1H), 7.93–7.74 (m, 2H), 7.59 (d, *J* = 7.8 Hz, 1H), 7.52–7.23 (m, 5H), 7.19–6.89 (m, 2H), 6.43–6.25 (m, 2H), 6.25–6.12 (m, 1H), 6.06–5.80 (m, 2H). ^13^C NMR (75 MHz, DMSO-*d*_6_) *δ* 153.2 142.6, 142.2, 137.5, 131.4, 130.8, 128.9, 128.1, 127.7, 127.2, 125.4, 122.3, 119.6, 119.1, 112.0, 110.8, 107.5, 106.1, 45.2, 31.2. IR (KBr) *ν* 2929, 1636, 1459, 1328, 1148, 736, 696 cm^−1^; HRMS: *m*/*z* calcd for ([C_22_H_16_N_4_O + H]^+^): 353.1397; found: 353.1399.

#### 3′-Phenyl-9′,10′-dihydrospiro[fluorene-9,4′-[1,2,3]triazolo[1′,5′:1,6]pyrido[3,4-*b*]indole] (3ap)

White solid (114 mg, 52%); mp 204–205 °C; ^1^H NMR (300 MHz, DMSO-*d*_6_) *δ* 11.46 (s, 1H), 7.76 (d, *J* = 7.5 Hz, 2H), 7.39–7.19 (m, 3H), 7.12–6.96 (m, 5H), 6.94–6.75 (m, 3H), 6.55–6.40 (m, 1H), 6.33 (d, *J* = 7.1 Hz, 2H), 6.22 (s, 2H), 5.99 (d, *J* = 8.0 Hz, 1H). ^13^C NMR (75 MHz, DMSO-*d*_6_) *δ* 149.7, 142.8, 140.9, 137.8, 133.9, 131.3, 129.1, 128.8, 128.7, 128.5, 127.8, 127.6, 125.3, 123.8, 122.0, 120.7, 119.4, 118.3, 112.0, 108.1, 50.8, 45.5. IR (KBr) *ν* 2932, 1721, 1448, 1245, 743, 696 cm^−1^; HRMS: *m*/*z* calcd for ([C_30_H_20_N_4_ + H]^+^): 473.1761; found: 473.1755.

#### 3′-(*p*-Tolyl)-9′,10′-dihydrospiro[fluorene-9,4′-[1,2,3]triazolo[1′,5′:1,6]pyrido[3,4-*b*]indole] (3aq)

White solid (119 mg, 53%); mp 340–341 °C; ^1^H NMR (300 MHz, DMSO-*d*_6_) *δ* 11.45 (s, 1H), 7.80 (d, *J* = 7.6 Hz, 2H), 7.44–7.20 (m, 3H), 7.18–6.98 (m, 4H), 6.96–6.79 (m, 1H), 6.66 (d, *J* = 7.9 Hz, 2H), 6.57–6.41 (m, 1H), 6.37–6.11 (m, 4H), 5.99 (d, *J* = 7.9 Hz, 1H), 2.09 (s, 3H). ^13^C NMR (75 MHz, DMSO-*d*_6_) *δ* 149.7, 142.6, 140.9, 137.7, 136.8, 133.6, 129.0, 128.7, 128.5, 128.4, 128.2, 125.2, 123.8, 121.9, 120.7, 119.3, 118.3, 111.9, 108.1, 50.8, 45.4, 21.2. IR (KBr) *ν* 2935, 1643, 1597, 1112, 1010, 743, 624 cm^−1^; HRMS: *m*/*z* calcd for ([C_31_H_22_N_4_ + H]^+^): 451.1917; found: 451.1919.

#### 3′-(4-Methoxyphenyl)-9′,10′-dihydrospiro[fluorene-9,4′-[1,2,3]triazolo[1′,5′:1,6]pyrido[3,4-*b*]indole] (3ar)

White solid (131 mg, 56%); mp 313–314 °C; ^1^H NMR (300 MHz, DMSO-*d*_6_) *δ* 11.47 (s, 1H), 7.82 (d, *J* = 7.5 Hz, 2H), 7.40–7.24 (m, 3H), 7.16–7.00 (m, 4H), 6.97–6.83 (m, 1H), 6.58–6.46 (m, 1H), 6.47–6.34 (m, 2H), 6.34–6.13 (m, 4H), 6.00 (d, *J* = 8.0 Hz, 1H), 3.61 (s, 3H). ^13^C NMR (75 MHz, DMSO-*d*_6_) *δ* 158.8, 149.7, 142.4, 140.9, 137.7, 133.5, 129.8, 129.1, 128.8, 128.5, 125.2, 123.8, 123.7, 121.9, 120.7, 119.3, 118.3, 113.1, 112.0, 108.1, 55.4, 50.7, 45.4. IR (KBr) *ν* 2936, 1614, 1506, 1448, 1249, 1184, 830, 743 cm^−1^; HRMS: *m*/*z* calcd for ([C_31_H_22_N_4_ + H]^+^): 467.1866; found: 467.1865.

#### 3′-(4-Chlorophenyl)-9′,10′-dihydrospiro[fluorene-9,4′-[1,2,3]triazolo[1′,5′:1,6]pyrido[3,4-*b*]indole] (3as)

White solid (120 mg, 51%); mp 328–329 °C; ^1^H NMR (300 MHz, DMSO-*d*_6_) *δ* 11.47 (s, 1H), 7.81 (d, *J* = 7.6 Hz, 2H), 7.40–7.22 (m, 3H), 7.18–6.98 (m, 4H), 6.98–6.83 (m, 3H), 6.58–6.40 (m, 1H), ^1^H NMR (300 MHz, DMSO) *δ* 11.47 (s, 1H), 7.81 (d, *J* = 7.6 Hz, 2H), 7.40–7.22 (m, 3H), 7.18–6.98 (m, 4H), 6.98–6.83 (m, 3H), 6.50 (t, *J* = 7.5 Hz, 1H), 6.37–6.26 (m, 2H), 6.22 (s, 2H), 5.99 (d, *J* = 8.0 Hz, 1H). ^13^C NMR (75 MHz, DMSO-*d*_6_) *δ* 149.5, 141.5, 140.8, 137.8, 134.3, 132.6, 130.4, 130.2, 129.1, 129.0, 128.7, 127.7, 125.3, 123.8, 122.0, 120.8, 119.5, 118.3, 112.0, 107.8, 50.7, 45.5. IR (KBr) *ν* 2933, 1632, 1448, 1328, 1090, 743 cm^−1^; HRMS: *m*/*z* calcd for ([C_31_H_21_ClN_4_ + H]^+^): 485.1528; found: 485.1529.

#### 9-Methyl-3,4,4-triphenyl-9,10-dihydro-4*H*-[1,2,3]triazolo[1′,5′:1,6]pyrido[3,4-*b*]indole (3ba)

White solid (68 mg, 30%); mp 295–296 °C; ^1^H NMR (300 MHz, DMSO) *δ* 7.46 (d, *J* = 8.3 Hz, 1H), 7.19–6.95 (m, 14H), 6.84–6.70 (m, 3H), 6.52 (d, *J* = 8.0 Hz, 1H), 5.98 (s, 2H), 3.79 (s, 3H). ^13^C NMR (75 MHz, DMSO) *δ* 144.3, 143.2, 138.4, 137.7, 132.1, 130.6, 129.6, 129.1, 128.4, 127.9, 127.8, 127.4, 124.6, 121.8, 120.1, 120.0, 114.6, 110.5, 51.4, 44.4, 30.2. IR (KBr) *ν* 2929, 1636, 1597, 1361, 1010, 754, 700 cm^−1^; HRMS: *m*/*z* calcd for ([C_31_H_24_N_4_ + H]^+^): 453.2074; found: 453.2075.

#### 9-Methyl-4,4-diphenyl-3-(*p*-tolyl)-9,10-dihydro-4*H*-[1,2,3]triazolo[1′,5′:1,6]pyrido[3,4-*b*]indole (3bb)

White solid (84 mg, 36%); mp 340–341 °C; ^1^H NMR (300 MHz, DMSO-*d*_6_) *δ* 7.47 (d, *J* = 8.3 Hz, 1H), 7.21–6.94 (m, 11H), 6.92–6.70 (m, 3H), 6.61 (d, *J* = 8.0 Hz, 2H), 6.51 (d, *J* = 7.9 Hz, 1H), 5.97 (s, 2H), 3.79 (s, 3H), 2.18 (s, 3H). ^13^C NMR (75 MHz, DMSO-*d*_6_) *δ* 144.2, 143.3, 138.4, 137.6, 137.1, 130.5, 129.4, 129.3, 129.1, 128.4, 127.4, 124.6, 121.8, 120.0, 119.9, 114.4, 110.5, 51.4, 44.4, 30.2, 21.2. IR (KBr) *ν* 2931, 1636, 1590, 1318, 1007, 746, 700 cm^−1^; HRMS: *m*/*z* calcd for ([C_32_H_26_N_4_ + H]^+^): 467.2230; found: 467.2230.

#### 3-(4-Methoxyphenyl)-9-methyl-4,4-diphenyl-9,10-dihydro-4*H*-[1,2,3]triazolo[1′,5′:1,6]pyrido[3,4-*b*]indole (3bc)

White solid (59 mg, 24%); mp 317–318 °C; ^1^H NMR (300 MHz, DMSO-*d*_6_) *δ* 7.49 (d, *J* = 8.3 Hz, 1H), 7.19–7.00 (m, 11H), 6.80 (t, *J* = 7.6 Hz, 1H), 6.70–6.50 (m, 5H), 5.99 (s, 2H), 3.81 (s, 3H), 3.67 (s, 3H). ^13^C NMR (75 MHz, DMSO-*d*_6_) *δ* 159.0, 144.0, 143.3, 138.4, 137.4, 130.8, 130.5, 129.1, 128.4, 127.4, 124.6, 124.4, 121.8, 120.1, 119.9, 114.5, 113.3, 110.5, 55.5, 51.4, 44.4, 30.2. IR (KBr) *ν* 2933, 2836, 1614, 1498, 1318, 1249, 1177, 833, 703 cm^−1^; HRMS: *m*/*z* calcd for ([C_32_H_26_N_4_O + H]^+^): 483.2179; found: 483.2176.

#### 9-Methyl-3-phenyl-4,4-di-*p*-tolyl-9,10-dihydro-4*H*-[1,2,3]triazolo[1′,5′:1,6]pyrido[3,4-*b*]indole (3be)

White solid (84 mg, 35%); mp 281–282 °C; ^1^H NMR (300 MHz, DMSO-*d*_6_) *δ* 7.45 (d, *J* = 8.0 Hz, 1H), 7.20–6.94 (m, 5H), 6.89 (s, 8H), 6.83–6.71 (m, 3H), 6.57 (d, *J* = 8.0 Hz, 1H), 5.95 (s, 2H), 3.78 (s, 3H), 2.16 (s, 6H). ^13^C NMR (75 MHz, DMSO-*d*_6_) *δ* 144.2, 140.5, 138.4, 138.0, 136.4, 132.2, 130.3, 129.6, 128.9, 127.8, 127.7, 124.6, 121.7, 120.2, 119.9, 114.8, 110.4, 50.8, 44.4, 30.2, 20.9. IR (KBr) *ν* 2934, 1636, 1509, 1361, 1010, 761, 700 cm^−1^; HRMS: *m*/*z* calcd for ([C_33_H_28_N_4_ + H]^+^): 481.2387; found: 481.2388.

#### 4,9-Dimethyl-3,4-diphenyl-9,10-dihydro-4*H*-[1,2,3]triazolo[1′,5′:1,6]pyrido[3,4-*b*]indole (3bh)

White solid (117 mg, 60%); mp 244–245 °C; ^1^H NMR (300 MHz, DMSO-*d*_6_) *δ* 7.44 (d, *J* = 8.2 Hz, 1H), 7.34–7.12 (m, 9H), 7.08 (t, *J* = 7.6 Hz, 1H), 6.85 (t, *J* = 7.5 Hz, 1H), 6.81–6.72 (m, 2H), 6.11 (AB, *J* = 17.0 Hz, 2H), 3.78 (s, 3H), 1.91 (s, 3H). ^13^C NMR (75 MHz, DMSO-*d*_6_) *δ* 146.8, 142.6, 139.0, 138.6, 132.5, 129.3, 128.7, 128.3, 127.6, 127.5, 127.0, 123.7, 121.8, 119.5, 113.5, 110.2, 44.4, 40.8, 30.1, 25.0. IR (KBr) *ν* 2936, 1639, 1491, 1325, 1090, 739, 703 cm^−1^; HRMS: *m*/*z* calcd for ([C_26_H_22_N_4_ + H]^+^): 391.1917; found: 391.1919.

#### 4-(4-Bromophenyl)-4,9-dimethyl-3-phenyl-9,10-dihydro-4*H*-[1,2,3]triazolo[1′,5′:1,6]pyrido[3,4-*b*]indole (3bj)

White solid (134 mg, 57%); mp 295–296 °C; ^1^H NMR (300 MHz, DMSO-*d*_6_) *δ* 7.45 (d, *J* = 8.2 Hz, 1H), 7.42–7.33 (m, 2H), 7.33–7.04 (m, 7H), 6.93–6.74 (m, 3H), 6.10 (AB, 17.0 Hz, 2H), 3.78 (s, 3H), 1.89 (s, 3H). ^13^C NMR (75 MHz, DMSO-*d*_6_) *δ* 146.0, 142.7, 138.6, 138.4, 132.4, 131.4, 129.9, 129.4, 128.4, 127.8, 123.5, 122.0, 120.1, 119.6, 119.2, 112.9, 110.3, 44.4, 40.8, 30.1, 25.2. IR (KBr) *ν* 2932, 1636, 1491, 1321, 1007, 739, 700 cm^−1^; HRMS: *m*/*z* calcd for ([C_26_H_21_N_4_Br + H]^+^): 469.1022; found: 469.1025.

#### 9-Methyl-3,4-diphenyl-9,10-dihydro-4*H*-[1,2,3]triazolo[1′,5′:1,6]pyrido[3,4-*b*]indole (3bl)

White solid (77 mg, 41%); mp 272–273 °C; ^1^H NMR (300 MHz, DMSO-*d*_6_) *δ* 7.89–7.67 (m, 2H), 7.62–7.43 (m, 2H), 7.43–7.20 (m, 5H), 7.22–7.07 (m, 3H), 7.07–6.90 (m, 2H), 6.26–6.02 (m, 3H), 3.81 (s, 3H). ^13^C NMR (75 MHz, DMSO-*d*_6_) *δ* 143.0, 141.9, 138.4, 133.6, 131.5, 128.8, 128.8, 128.6, 128.4, 128.0, 127.3, 127.1, 124.8, 122.1, 119.6, 119.3, 110.1, 108.7, 44.7, 39.2, 37.2, 30.2. IR (KBr) *ν* 2934, 1632, 1470, 1321, 1007, 750, 696 cm^−1^; HRMS: *m*/*z* calcd for ([C_25_H_20_N_4_ + H]^+^): 377.1761; found: 377.1759.

#### 4-(Furan-2-yl)-4,9-dimethyl-3-phenyl-9,10-dihydro-4*H*-[1,2,3]triazolo[1′,5′:1,6]pyrido[3,4-*b*]indole (3bo)

White solid (88 mg, 48%); mp 251–252 °C; ^1^H NMR (300 MHz, DMSO-*d*_6_) *δ* 7.91–7.74 (m, 2H), 7.61 (d, *J* = 7.8 Hz, 1H), 7.43 (m, *J* = 13.1, 11.3, 7.3 Hz, 3H), 7.36–7.24 (m, 2H), 7.21–7.10 (m, 1H), 7.08–6.96 (m, 1H), 6.42–6.28 (m, 2H), 6.26–6.13 (m, 1H), 6.13–5.88 (m, 2H), 3.77 (s, 3H). ^13^C NMR (75 MHz, DMSO-*d*_6_) *δ* 153.1, 142.6, 142.2, 138.2, 131.4, 130.6, 129.2, 128.9, 128.1, 127.2, 125.0, 122.2, 119.8, 119.2, 110.8, 110.2, 107.5, 105.5, 44.6, 31.2, 30.2. IR (KBr) *ν* 2933, 1636, 1473, 1365, 1007, 743, 689 cm^−1^; HRMS: *m*/*z* calcd for ([C_24_H_20_N_4_O + H]^+^): 367.1553; found: 367.1550.

#### 9′-Methyl-3′-phenyl-9′,10′-dihydrospiro[fluorene-9,4′-[1,2,3]triazolo[1′,5′:1,6]pyrido[3,4-*b*]indole] (3bp)

White solid (113 mg, 50%); mp 271–272 °C; ^1^H NMR (300 MHz, CDCl_3_) *δ* 7.69–7.45 (m, 2H), 7.35–7.18 (m, 3H), 7.13–6.97 (m, 4H), 6.96–6.88 (m, 2H), 6.89–6.77 (m, 2H), 6.73–6.57 (m, 1H), 6.48–6.32 (m, 2H), 6.24 (d, *J* = 8.0 Hz, 1H), 6.04 (s, 2H), 3.79 (s, 3H). ^13^C NMR (75 MHz, CDCl_3_) *δ* 148.8, 143.6, 140.6, 138.3, 134.0, 130.3, 128.8, 128.2, 128.0, 127.9, 127.2, 127.0, 124.6, 123.4, 122.2, 119.9, 119.8, 119.2, 108.9, 108.8, 50.5, 44.5, 29.9. IR (KBr) *ν* 2933, 1610, 1477, 1321, 1007, 902, 746, 696 cm^−1^; HRMS: *m*/*z* calcd for ([C_31_H_22_N_4_ + H]^+^): 451.1917; found: 451.1912.

#### 9′-Methyl-3′-(*p*-tolyl)-9′,10′-dihydrospiro[fluorene-9,4′-[1,2,3]triazolo[1′,5′:1,6]pyrido[3,4-*b*]indole] (3bq)

White solid (118 mg, 51%); mp 352–353 °C; ^1^H NMR (300 MHz, DMSO-*d*_6_) *δ* 7.80 (d, *J* = 7.6 Hz, 2H), 7.40 (d, *J* = 8.3 Hz, 1H), 7.35–7.21 (m, 2H), 7.16–6.89 (m, 5H), 6.66 (d, *J* = 7.8 Hz, 2H), 6.61–6.47 (m, 1H), 6.39–6.17 (m, 4H), 6.01 (d, *J* = 7.9 Hz, 1H), 3.84 (s, 3H), 2.10 (s, 3H). ^13^C NMR (75 MHz, DMSO-*d*_6_) *δ* 149.7, 142.6, 140.9, 138.5, 136.8, 133.5, 130.5, 128.8, 128.5, 128.4, 128.2, 125.2, 123.4, 121.9, 120.7, 119.6, 118.3, 110.2, 107.5, 50.8, 44.8, 30.3, 21.2. IR (KBr) *ν* 2934, 1618, 1479, 1321, 1007, 743 cm^−1^; HRMS: *m*/*z* calcd for ([C_32_H_24_N_4_ + H]^+^): 465.2074; found: 465.2067.

#### 4-(4-Bromophenyl)-7-chloro-4-methyl-3-phenyl-9,10-dihydro-4*H*-[1,2,3]triazolo[1′,5′:1,6]pyrido[3,4-*b*]indole (3cj)

White solid (113 mg, 46%); mp 297–298 °C; ^1^H NMR (400 MHz, DMSO-*d*_6_) *δ* 11.55 (s, 1H), 7.48 (s, 1H), 7.41 (d, *J* = 8.3 Hz, 2H), 7.37–7.30 (m, 1H), 7.30–7.18 (m, 4H), 7.18–7.09 (m, 1H), 6.98–6.75 (m, 3H), 6.01 (AB, 17.0 Hz, 2H), 1.90 (s, 3H). ^13^C NMR (100 MHz, DMSO-*d*_6_) *δ* 146.0, 143.1, 138.7, 138.6, 132.5, 131.9, 130.2, 129.7, 128.9, 128.8, 127.7, 127.2, 123.0, 120.7, 120.6, 120.2, 114.2, 112.2, 45.3, 40.7, 25.4. IR (KBr) *ν* 2933, 1627, 1487, 1383, 1326, 1008, 766, 700 cm^−1^; HRMS: *m*/*z* calcd for ([C_25_H_18_N_4_BrCl + H]^+^): 489.0476; found: 489.0480.

#### 3,3,5-Triphenyl-3,10-dihydroazepino[3,4-*b*]indole (4aa)^5^

White solid (109 mg, 53%); mp 276–277 °C; ^1^H NMR (300 MHz, CDCl_3_) *δ* 8.79 (s, 1H), 8.45 (s, 1H), 7.65–7.43 (m, 6H), 7.42–7.29 (m, 3H), 7.24–7.07 (m, 6H), 7.07–6.96 (m, 2H), 6.96–6.81 (m, 1H), 6.75 (d, *J* = 7.8 Hz, 1H), 5.73 (s, 1H). ^13^C NMR (75 MHz, CDCl_3_) *δ* 150.8, 148.0, 140.7, 138.2, 136.0, 135.8, 129.1, 128.2, 128.0, 128.0, 127.6, 127.2, 126.2, 124.9, 124.6, 122.9, 120.8, 120.0, 111.3, 71.0. IR (KBr) *v* 3057, 1621, 1520, 1487, 1332, 1234, 750, 703 cm^−1^; HRMS: *m*/*z* calcd for ([C_30_H_22_N_2_ + H]^+^): 411.1856; found: 411.1858.

#### 3,3-Diphenyl-5-(*p*-tolyl)-3,10-dihydroazepino[3,4-*b*]indole (4ab)

White solid (93 mg, 44%); mp 292–293 °C; ^1^H NMR (300 MHz, CDCl_3_) *δ* 8.71 (s, 1H), 8.64 (s, 1H), 7.57–7.42 (m, 4H), 7.42–7.28 (m, 2H), 7.22–7.03 (m, 8H), 7.04–6.93 (m, 2H), 6.94–6.73 (m, 2H), 5.72 (s, 1H), 2.39 (s, 3H). ^13^C NMR (75 MHz, CDCl_3_) *δ* 151.0, 148.0, 138.1, 137.8, 136.0, 135.8, 129.0, 128.9, 127.6, 127.4, 127.2, 126.2, 124.8, 124.7, 122.9, 120.9, 119.9, 111.4, 71.1, 21.3. IR (KBr) *ν* 3055, 2836, 1614, 1506, 1448, 1253, 1184, 1018, 830, 743 cm^−1^; HRMS: *m*/*z* calcd for ([C_31_H_24_N_2_ + H]^+^): 425.2012; found: 425.2010.

#### 5-(4-Methoxyphenyl)-3,3-diphenyl-3,10-dihydroazepino[3,4-*b*]indole (4ac)^5^

White solid (132 mg, 60%); mp 293–294 °C; ^1^H NMR (300 MHz, CDCl_3_) *δ* 8.78 (s, 1H), 8.39 (s, 1H), 7.57–7.43 (m, 4H), 7.42–7.32 (m, 2H), 7.22–7.05 (m, 6H), 7.06–6.96 (m, 2H), 6.96–6.77 (m, 4H), 5.68 (s, 1H), 3.85 (s, 3H). ^13^C NMR (75 MHz, CDCl_3_) *δ* 159.6, 150.7, 148.1, 137.6, 136.1, 135.7, 133.3, 130.3, 127.6, 127.2, 127.0, 126.1, 124.9, 124.7, 123.0, 121.2, 120.0, 113.5, 111.3, 71.0, 55.3. IR (KBr) *ν* 3056, 2832, 1621, 1524, 1506, 1245, 1173, 1028, 754, 703 cm^−1^; HRMS: *m*/*z* calcd for ([C_31_H_24_N_2_O + H]^+^): 441.1961; found: 441.1965.

#### 3,3-Bis(4-methoxyphenyl)-5-phenyl-3,10-dihydroazepino[3,4-*b*]indole (4at)

White solid (122 mg, 52%); mp 160–161 °C; ^1^H NMR (300 MHz, CDCl_3_) *δ* 9.14 (s, 1H), 8.57 (s, 1H), 7.52–7.29 (m, 9H), 7.20–7.08 (m, 2H), 6.92–6.79 (m, 1H), 6.78–6.59 (m, 5H), 5.69 (s, 1H), 3.66 (s, 6H). ^13^C NMR (75 MHz, CDCl_3_) *δ* 157.6, 150.9, 140.8, 140.5, 138.0, 136.1, 136.0, 129.1, 128.5, 128.4, 128.2, 128.0, 124.8, 124.6, 122.8, 120.6, 119.9, 112.9, 111.5, 70.2, 55.1. IR (KBr) *ν* 3056, 2835, 1610, 1506, 1234, 1028, 750, 700 cm^−1^; HRMS: *m*/*z* calcd for ([C_32_H_26_N_2_O_2_ + H]^+^): 471.2067; found: 471.2062.

#### 10-Methyl-3,3,5-triphenyl-3,10-dihydroazepino[3,4-*b*]indole (4ba)

White solid (76 mg, 36%); mp 280–281 °C; ^1^H NMR (300 MHz, CDCl_3_) *δ* 8.89 (s, 1H), 7.63–7.43 (m, 6H), 7.42–7.30 (m, 3H), 7.24–7.18 (m, 2H), 7.17–7.05 (m, 4H), 7.05–6.94 (m, 2H), 6.91–6.81 (m, 1H), 6.75 (d, *J* = 8.2 Hz, 1H), 5.80 (s, 1H), 3.66 (s, 3H). ^13^C NMR (75 MHz, CDCl_3_) *δ* 149.7, 147.9, 140.8, 138.1, 138.0, 136.7, 129.2, 128.2, 128.0, 127.5, 127.1, 126.1, 124.5, 124.1, 122.8, 120.0, 119.7, 109.2, 71.1, 30.0. IR (KBr) *ν* 3055, 2825, 1639, 1618, 1130, 1235, 750, 616 cm^−1^; HRMS: *m*/*z* calcd for ([C_31_H_25_N_2_ + H]^+^): 425.2012; found: 425.2012.

#### 5-(4-Methoxyphenyl)-10-methyl-3,3-diphenyl-3,10-dihydroazepino[3,4-*b*]indole (4bc)

White solid (114 mg, 50%); mp 299–300 °C;^1^H NMR (300 MHz, CDCl_3_) *δ* 8.87 (s, 1H), 7.46 (d, *J* = 7.4 Hz, 4H), 7.42–7.35 (m, 2H), 7.27–7.13 (m, 2H), 7.13–7.03 (m, 4H), 7.02–6.93 (m, 2H), 6.93–6.79 (m, 4H), 5.74 (s, 1H), 3.83 (s, 3H), 3.62 (s, 3H). ^13^C NMR (75 MHz, CDCl_3_) *δ* 159.5, 149.6, 147.9, 138.1, 137.4, 136.7, 133.4, 130.2, 127.5, 127.1, 127.0, 126.0, 124.4, 124.2, 122.9, 120.2, 119.6, 113.5, 109.2, 71.1, 55.2, 30.0. IR (KBr) *ν* 3056, 2833, 1603, 1484, 1253, 1097, 736, 700 cm^−1^; HRMS: *m*/*z* calcd for ([C_32_H_26_N_2_O + H]^+^): 455.2118; found: 455.2189.

#### 10-Methyl-5-phenyl-3,3-di-*p*-tolyl-3,10-dihydroazepino[3,4-*b*]indole (4be)

White solid (122 mg, 54%); mp 303–304 °C;^1^H NMR (300 MHz, CDCl_3_) *δ* 8.87 (s, 1H), 7.52–7.41 (m, 2H), 7.39–7.27 (m, 7H), 7.27–7.13 (m, 2H), 6.95–6.80 (m, 5H), 6.74 (d, *J* = 8.1 Hz, 1H), 5.75 (d, *J* = 2.1 Hz, 1H), 3.66 (s, 3H), 2.15 (s, 6H). ^13^C NMR (75 MHz, CDCl_3_) *δ* 149.5, 145.2, 141.0, 137.9, 137.73, 136.8, 135.3, 129.2, 128.3, 128.2, 128.1, 127.9, 126.9, 124.3, 124.1, 122.9, 119.7, 119.6, 109.2, 70.7, 30.0, 20.9. IR (KBr) *ν* 3055, 2832, 1636, 1513, 1115, 1021, 754, 703 cm^−1^; HRMS: *m*/*z* calcd for ([C_33_H_28_N_2_ + H]^+^): 453.2325; found: 453.2323.

#### 3,3-Bis(4-methoxyphenyl)-10-methyl-5-phenyl-3,10-dihydroazepino[3,4-*b*]indole (4bt)

White solid (136 mg, 56%); mp 240–241 °C;^1^H NMR (300 MHz, CDCl_3_) *δ* 8.85 (s, 1H), 7.53–7.40 (m, 3H), 7.40–7.27 (m, 7H), 7.27–7.12 (m, 2H), 6.84 (s, 1H), 6.73 (d, *J* = 8.1 Hz, 1H), 6.68–6.60 (m, 3H), 5.72 (s, 1H), 3.65 (s, 3H), 3.64 (s, 6H). ^13^C NMR (75 MHz, CDCl_3_) *δ* 157.5, 149.4, 140.9, 140.4, 137.8, 137.7, 136.7, 129.1, 128.5, 128.1, 128.1, 127.9, 124.4, 124.1, 122.8, 119.6, 112.80, 109.3, 70.2, 55.0, 30.0. IR (KBr) *ν* 3056, 2828, 1603, 1502, 1238, 1169, 1032, 833, 750 cm^−1^; HRMS: *m*/*z* calcd for ([C_33_H_28_N_2_O_2_ + H]^+^): 485.2224; found: 485.2223.

## Conflicts of interest

There are no conflicts to declare.

## Supplementary Material

RA-011-D1RA03022A-s001

RA-011-D1RA03022A-s002
