# Supplementary material for: Synthesis of tetrahydro-β-carbolines from 2-indolylmethyl azides and propargylic alcohols
Source: RSC Adv. 2021 Jun 1;11(32):19639–46. doi: 10.1039/d1ra03022a (PMC9033608; doi:10.1039/d1ra03022a)
Supplement: RA-011-D1RA03022A-s001 [file RA-011-D1RA03022A-s001.pdf]

Electronic Supplementary Material (ESI) for RSC Advances.
This journal is © The Royal Society of Chemistry 2021

Electronic Supplementary Information

For

Synthesis of tetrahydro-*β*-carbolines from 2-indolylmethyl azides and propargylic alcohols

Haiting Yin,[a] Qin Ma,[a] Yushan Wang,[a] Xiaoxia Gu,[a] Zhijun Feng,[a] Yunjun Wu ,[a] Ming Wang,[a] and Shaoyin Wang *[a]
[a]Department of Chemistry, Institute of Synthesis and Application of Medical Materials, Chunhui Scientific Research Interest Group, Wannan Medical College, Wuhu, Anhui 241002, China.

E-mail: wsychem@163.com

Table of Contents

Copies of ^{1}H NMR and ^{13}C NMR S2-S39

Copies of ^{1}H NMR and ^{13}C NMR

DMSO-d_6, 300MHz

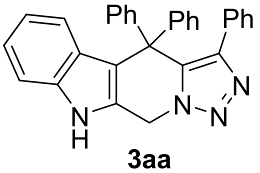

3aa

DMSO-d_6, 75MHz

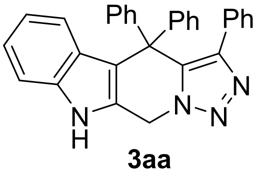

3aa

DMSO-d_6, 300MHz

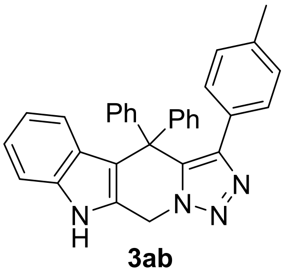

3ab

DMSO-d_6, 75MHz

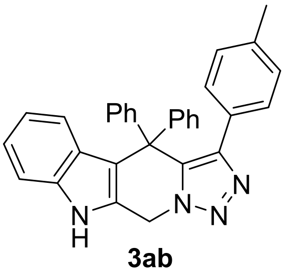

3ab

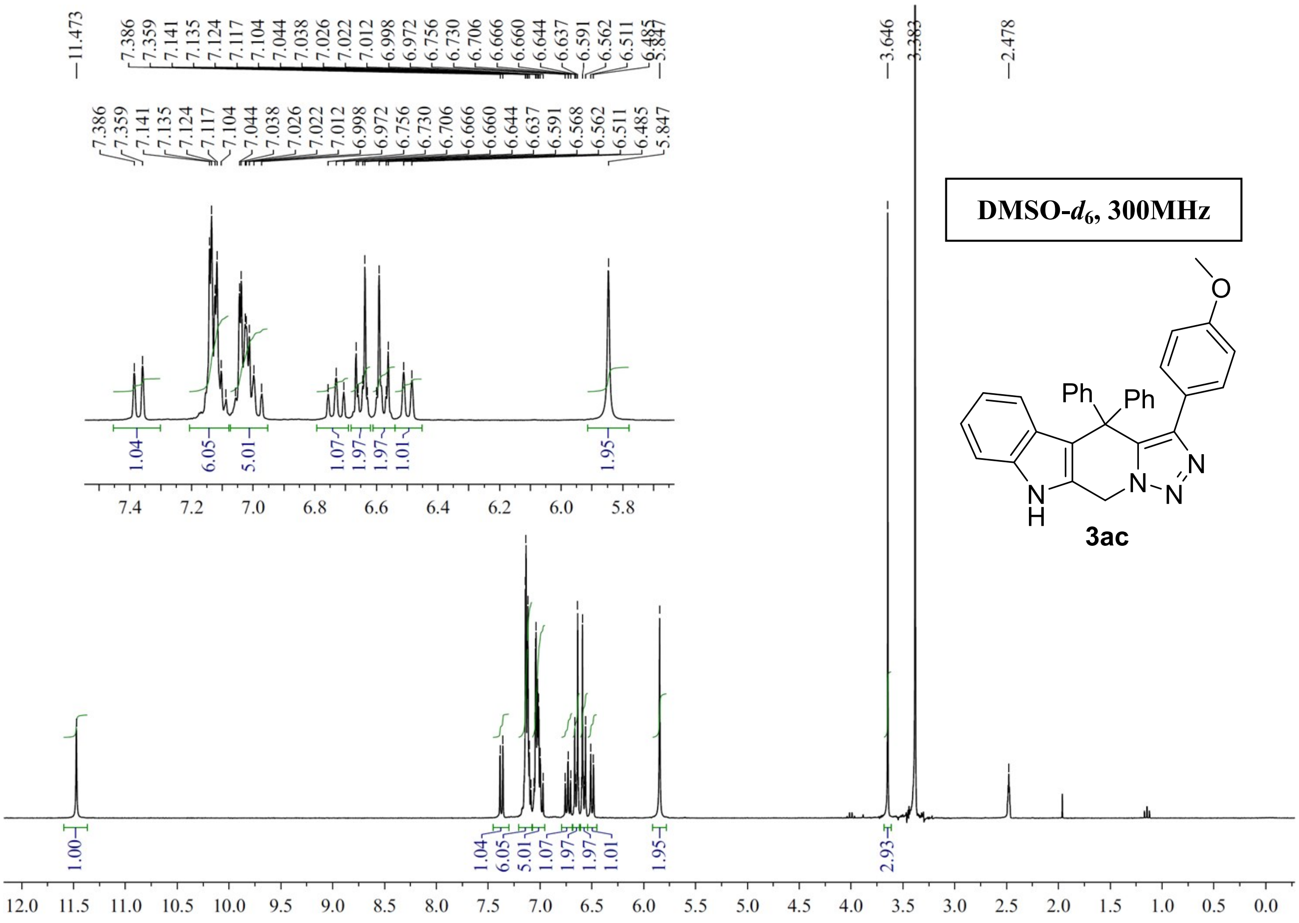
11.473
7.386 7.359 7.141 7.135 7.124 7.117 7.104 7.044 7.038 7.026 7.022 7.012 6.998 6.972 6.756 6.730 6.706 6.666 6.660 6.644 6.637 6.591 6.562 6.511 6.485 5.847
3.646
3.383
2.478
7.386 7.359 7.141 7.135 7.124 7.117 7.104 7.044 7.038 7.026 7.022 7.012 6.998 6.972 6.756 6.730 6.706 6.666 6.660 6.644 6.637 6.591 6.568 6.562 6.511 6.485 5.847
1.04 6.05 5.01 1.07 1.97 1.97 1.01 1.95
7.4 7.2 7.0 6.8 6.6 6.4 6.2 6.0 5.8
DMSO-d_6, 300MHz
Ph
Ph
O
N
N
N
H
3ac
1.00 1.04 6.05 5.01 1.07 1.97 1.97 1.01 1.95 2.93
12.0 11.5 11.0 10.5 10.0 9.5 9.0 8.5 8.0 7.5 7.0 6.5 6.0 5.5 5.0 4.5 4.0 3.5 3.0 2.5 2.0 1.5 1.0 0.5 0.0

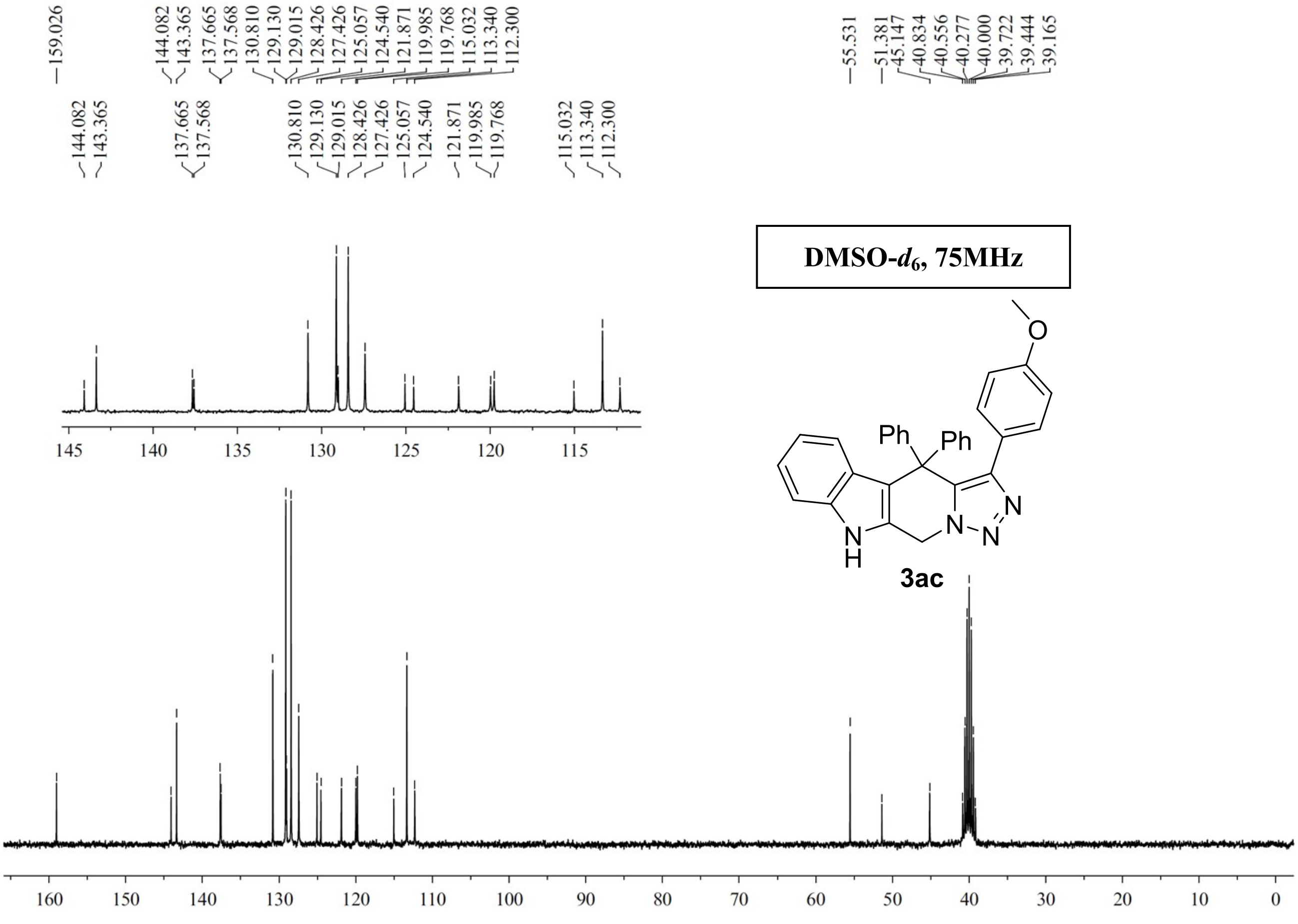
159.026
144.082 143.365 137.665 137.568 130.810 129.130 129.015 128.426 127.426 125.057 124.540 121.871 119.985 119.768 115.032 113.340 112.300
55.531 51.381 45.147 40.834 40.556 40.277 40.000 39.722 39.444 39.165
144.082 143.365 137.665 137.568 130.810 129.130 129.015 128.426 127.426 125.057 124.540 121.871 119.985 119.768 115.032 113.340 112.300
DMSO-d_6, 75MHz
145 140 135 130 125 120 115
Ph
Ph
O
N
N
N
H
3ac
160 150 140 130 120 110 100 90 80 70 60 50 40 30 20 10 0

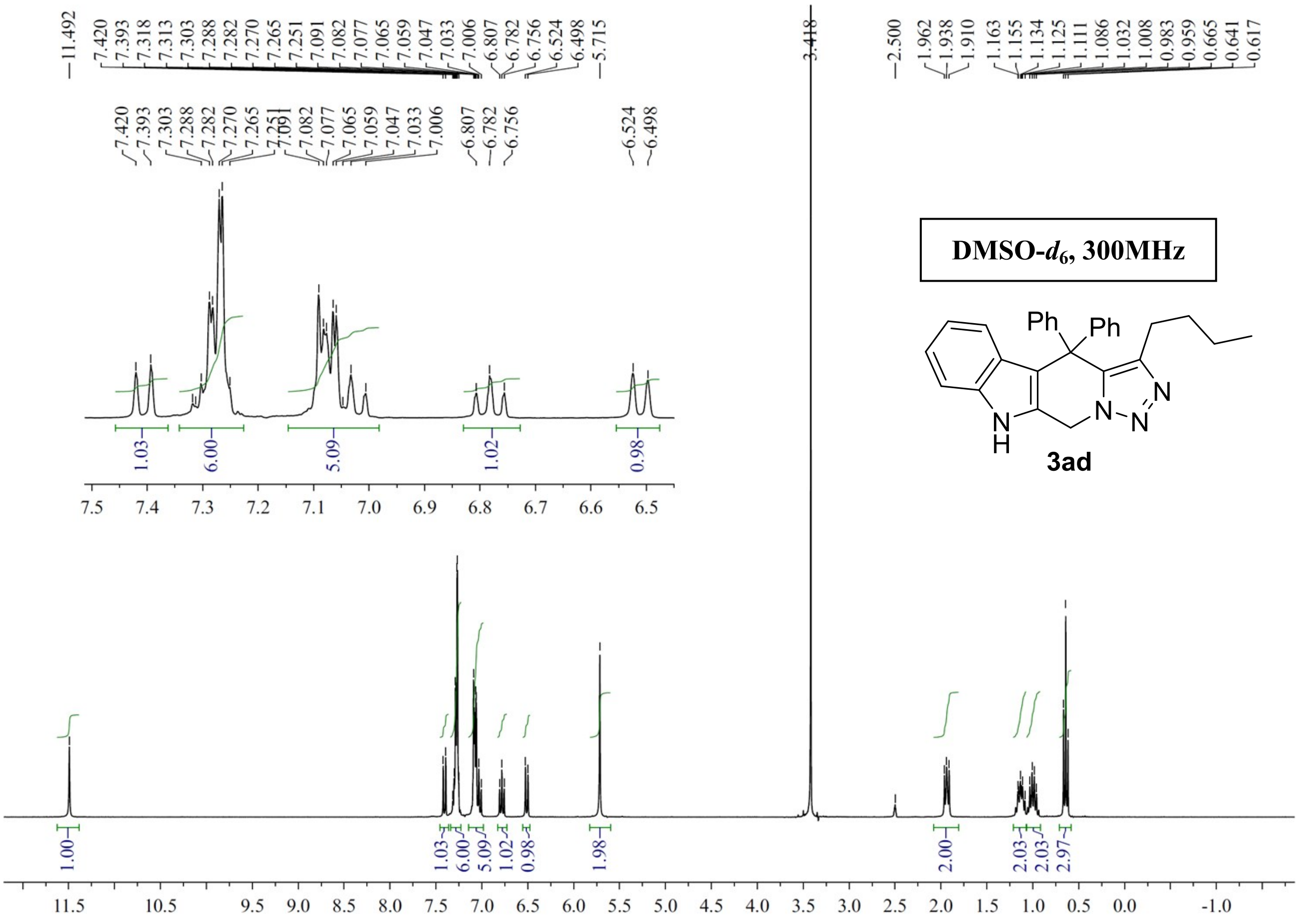
11.492
7.420 7.393 7.318 7.313 7.303 7.288 7.282 7.270 7.265 7.251 7.091 7.082 7.077 7.065 7.059 7.047 7.033 7.006 6.807 6.782 6.756 6.524 6.498
5.715
3.418
2.500
1.962 1.938 1.910
1.163 1.155 1.134 1.125 1.111 1.086 1.032 1.008 0.983 0.959 0.665 0.641 0.617
DMSO-d_6, 300MHz
Ph
Ph
N
N
N
H
3ad
1.03 6.00 5.09 1.02 0.98
1.00 1.03 6.00 5.09 1.02 0.98 1.98 2.00 2.03 2.03 2.97

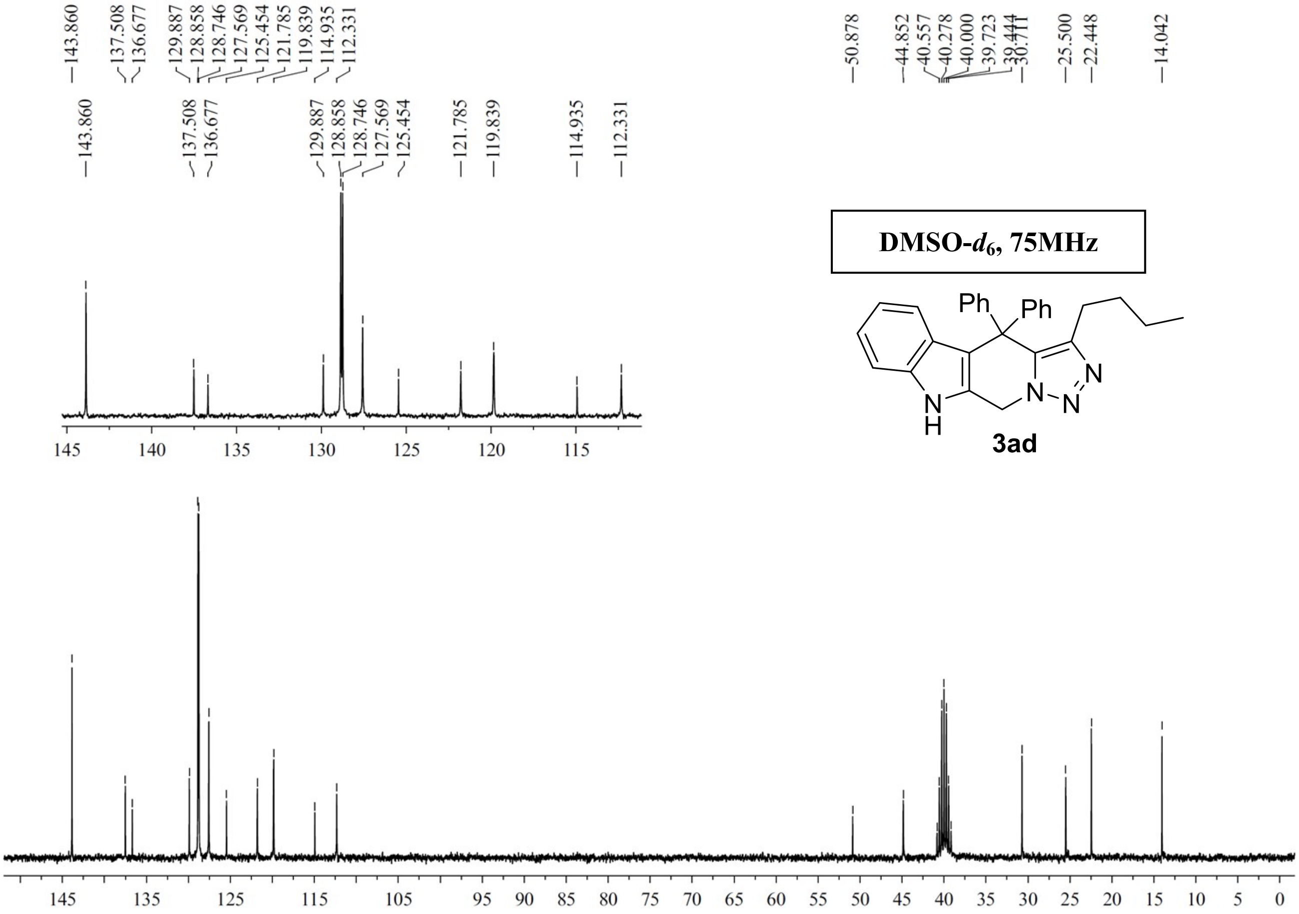
143.860
137.508 136.677
129.887 128.858 128.746 127.569 125.454 121.785 119.839 114.935 112.331
50.878
44.852 40.557 40.278 40.000 39.723 39.444 30.711
25.500
22.448
14.042
DMSO-d_6, 75MHz
Ph
Ph
N
N
N
H
3ad

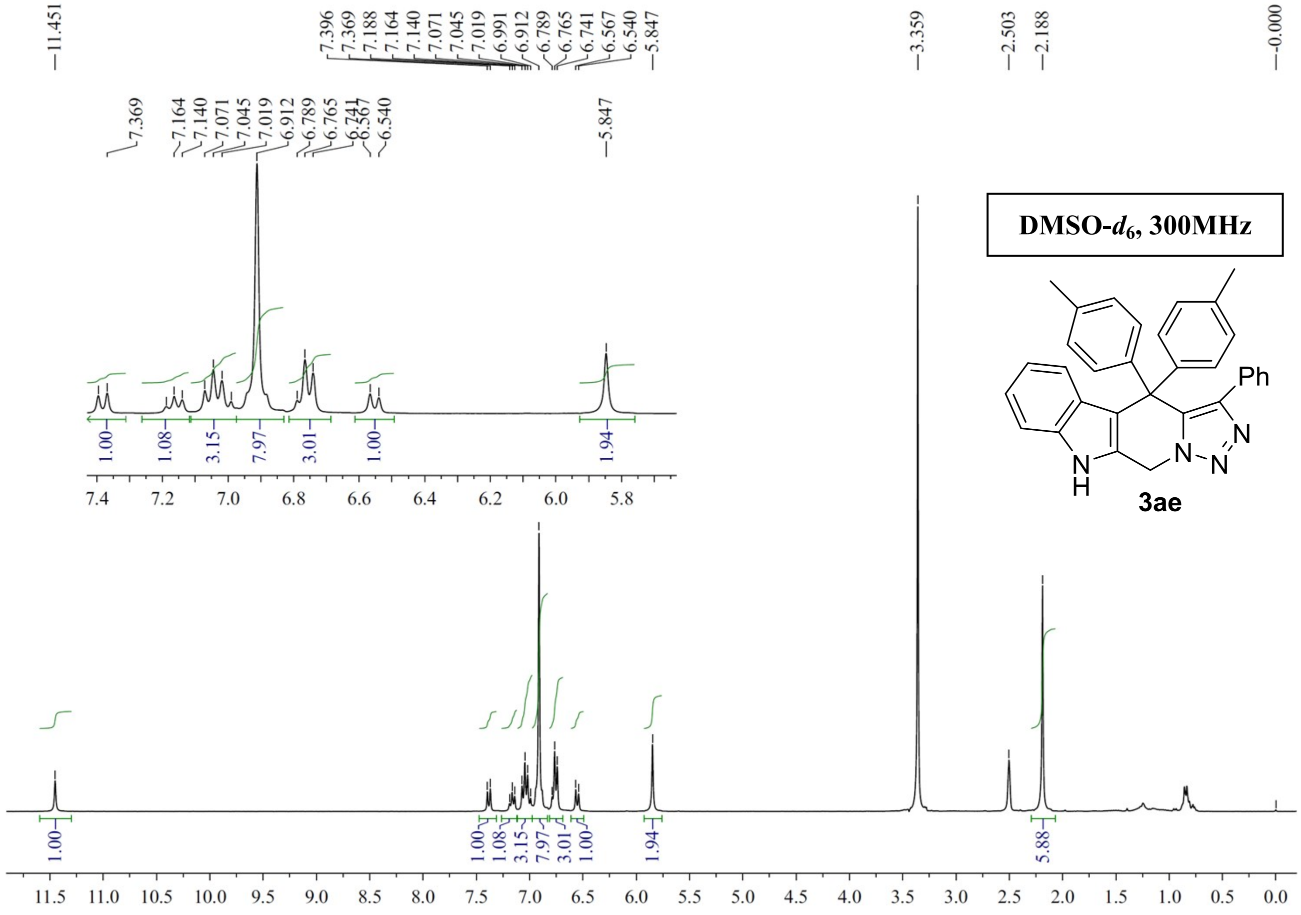
DMSO-d_6, 300MHz
Ph
N
N
N
NH
3ae

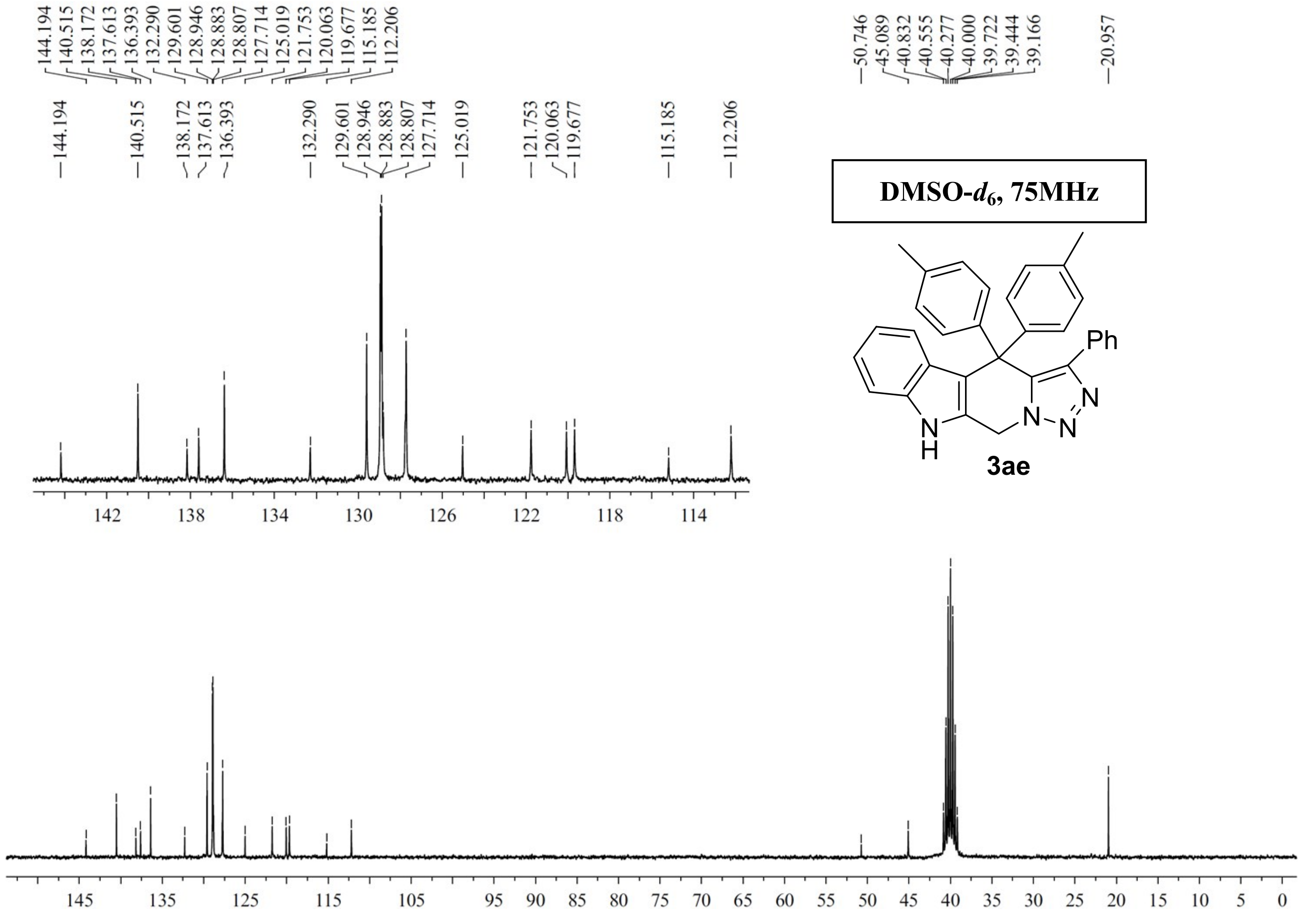
DMSO-d_6, 75MHz
Ph
N
N
N
NH
3ae

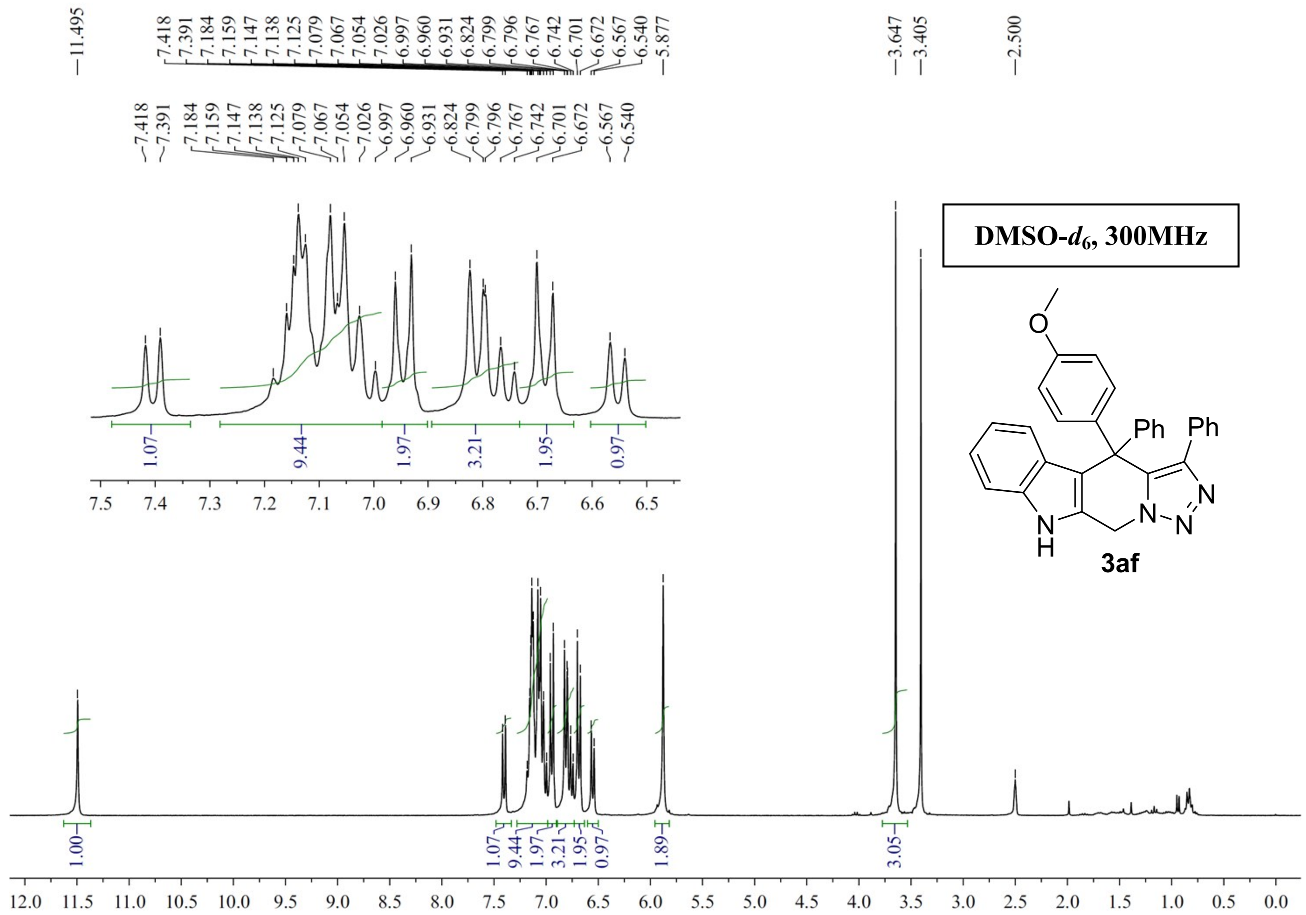
11.495
7.418 7.391 7.184 7.159 7.147 7.138 7.125 7.079 7.067 7.054 7.026 6.997 6.960 6.931 6.824 6.799 6.796 6.767 6.742 6.701 6.672 6.567 6.540 5.877
3.647
3.405
2.500
1.07 9.44 1.97 3.21 1.95 0.97
1.00 1.89 3.05
DMSO-d6, 300MHz
Ph
Ph
N
H
3af

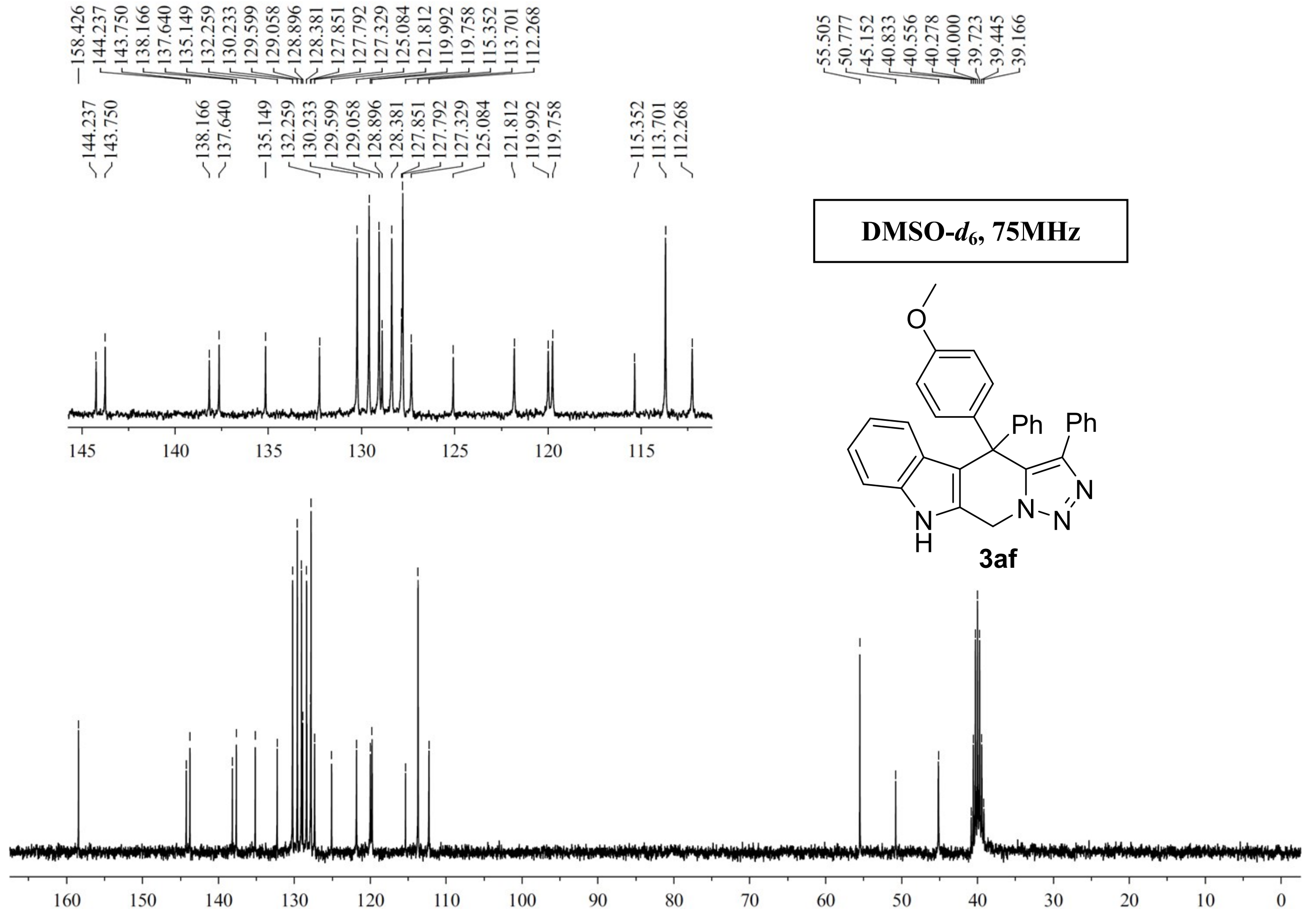
158.426 144.237 143.750 138.166 137.640 135.149 132.259 130.233 129.599 129.058 128.896 128.381 127.851 127.792 127.329 125.084 121.812 119.992 119.758 115.352 113.701 112.268
55.505 50.777 45.152 40.833 40.556 40.278 40.000 39.723 39.445 39.166
DMSO-d6, 75MHz
Ph
Ph
N
H
3af

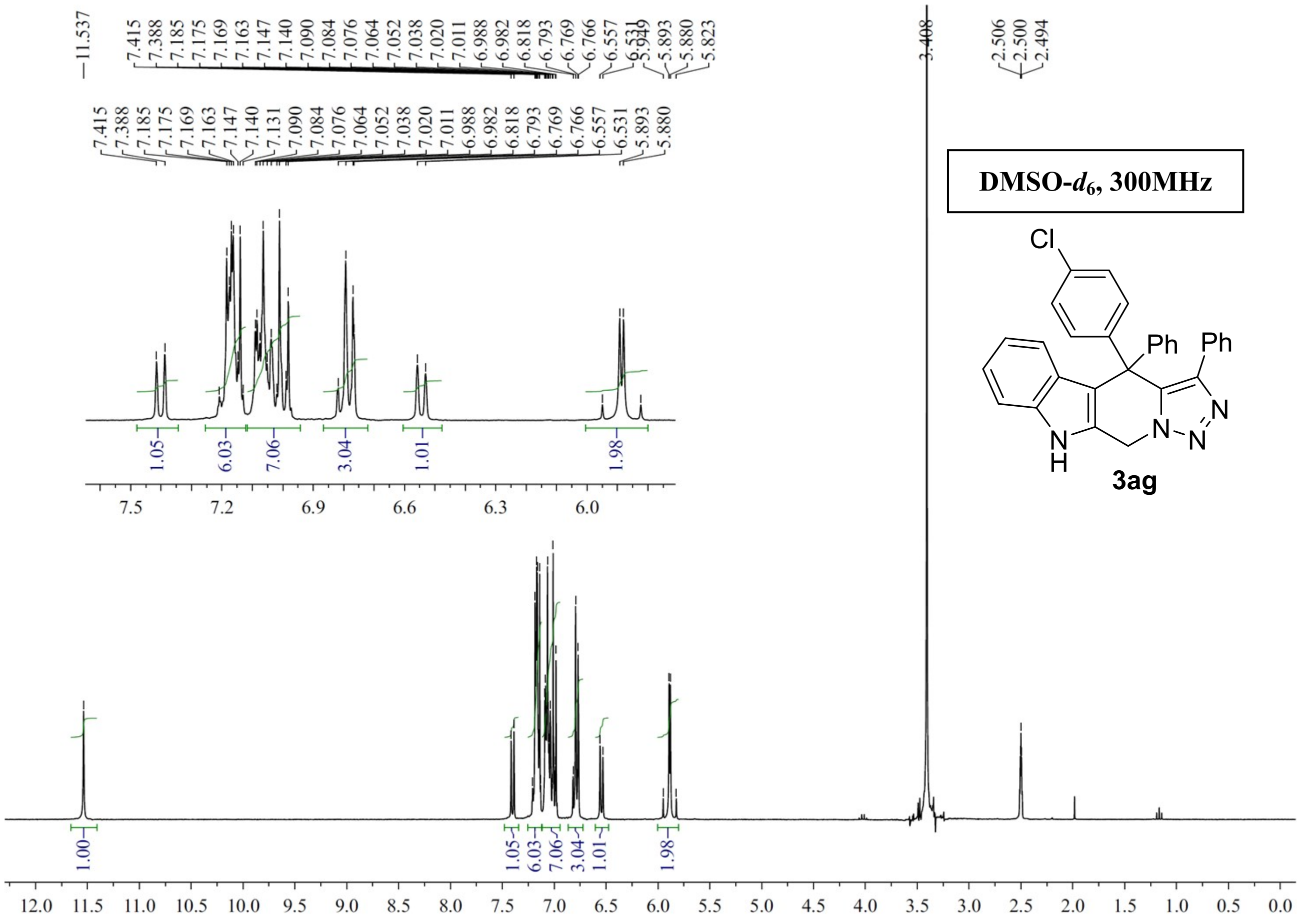
DMSO-d_6, 300MHz
Cl
Ph
Ph
N
N
N
N
H
3ag

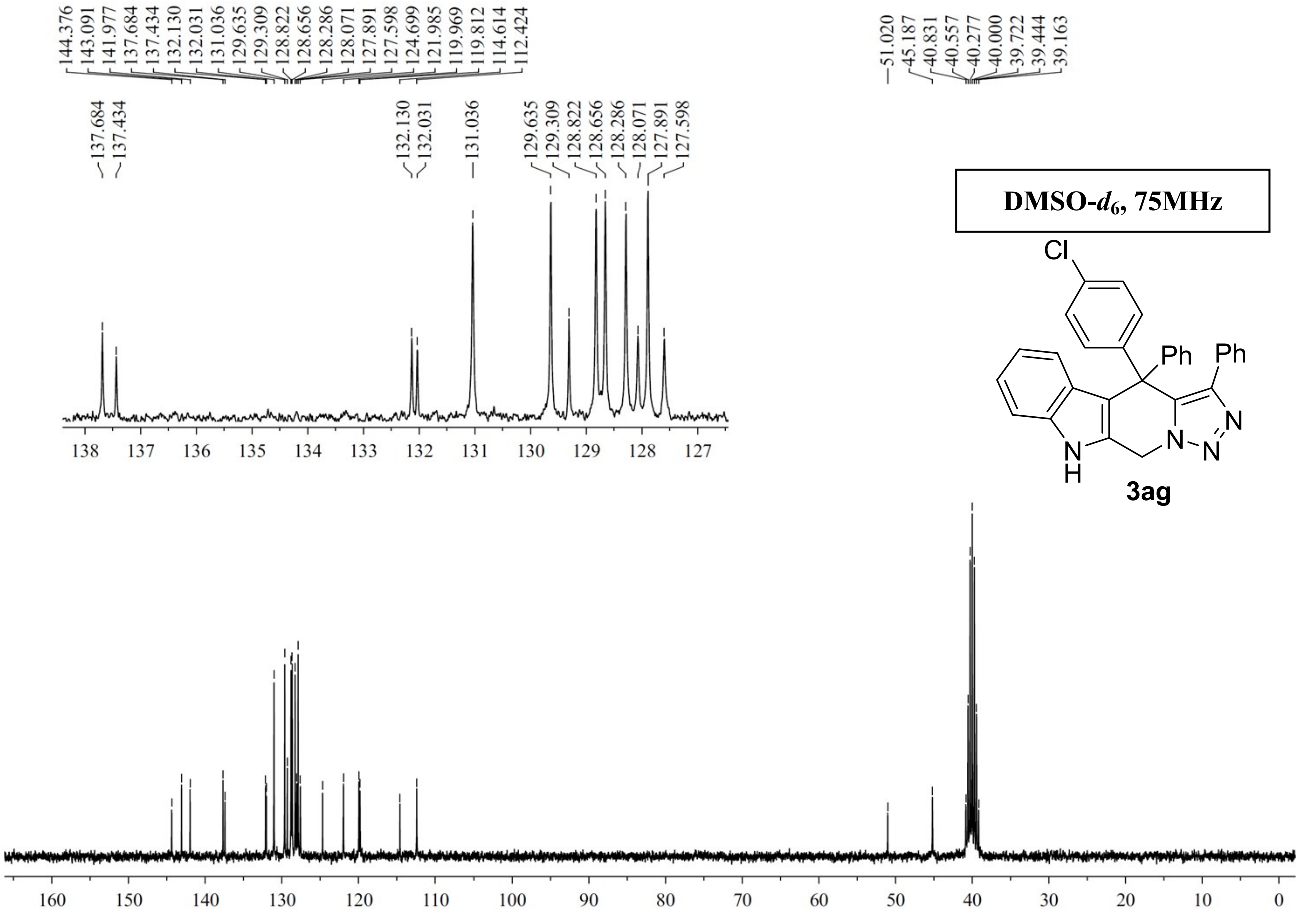
DMSO-d_6, 75MHz
Cl
Ph
Ph
N
N
N
N
H
3ag

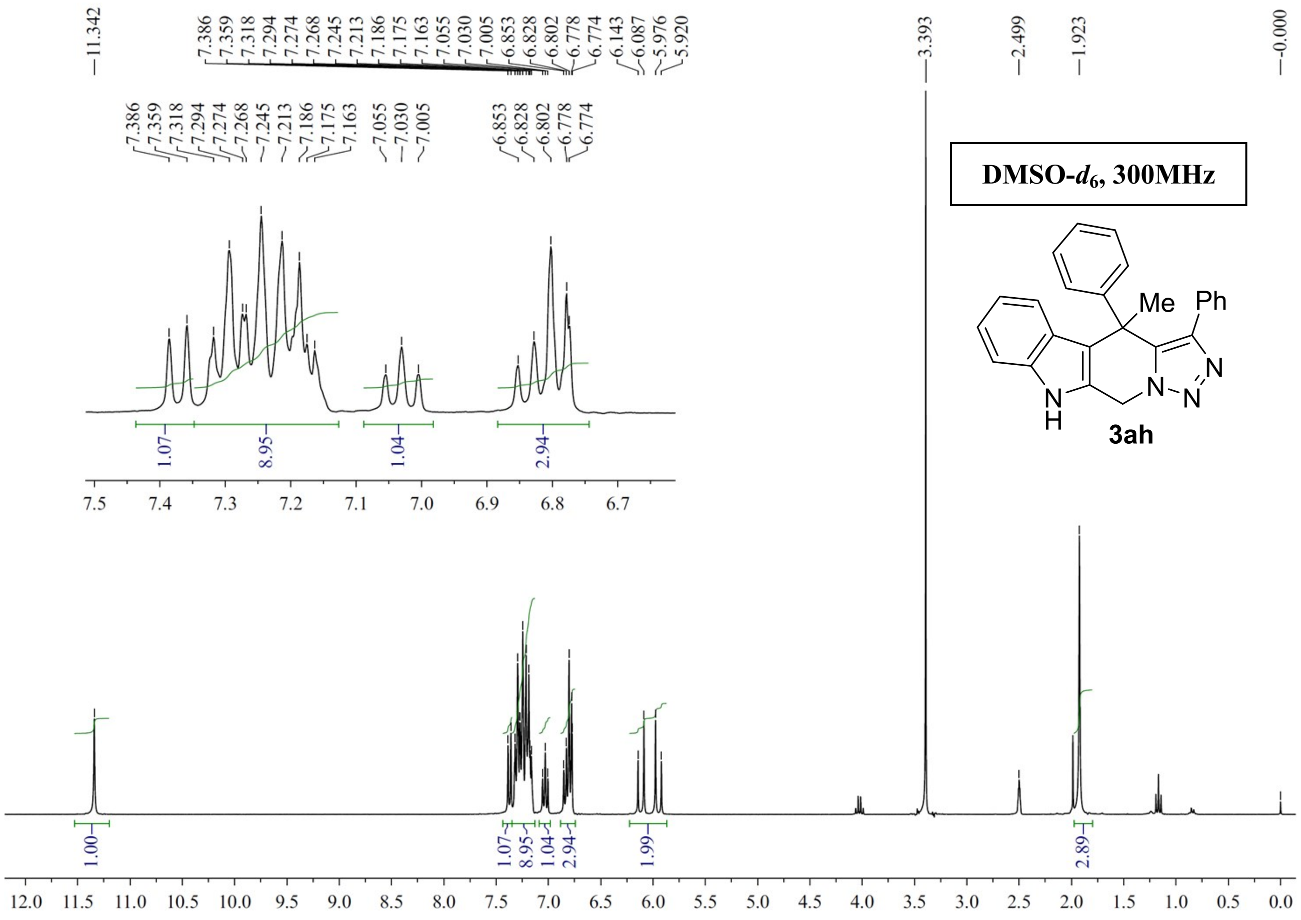
11.342
7.386 7.359 7.318 7.294 7.274 7.268 7.245 7.213 7.186 7.175 7.163 7.055 7.030 7.005 6.853 6.828 6.802 6.778 6.774 6.143 6.087 5.976 5.920
3.393
2.499
1.923
-0.000
DMSO-d6, 300MHz
Me
Ph
N
H
3ah
1.07
8.95
1.04
2.94
1.00
1.99
2.89

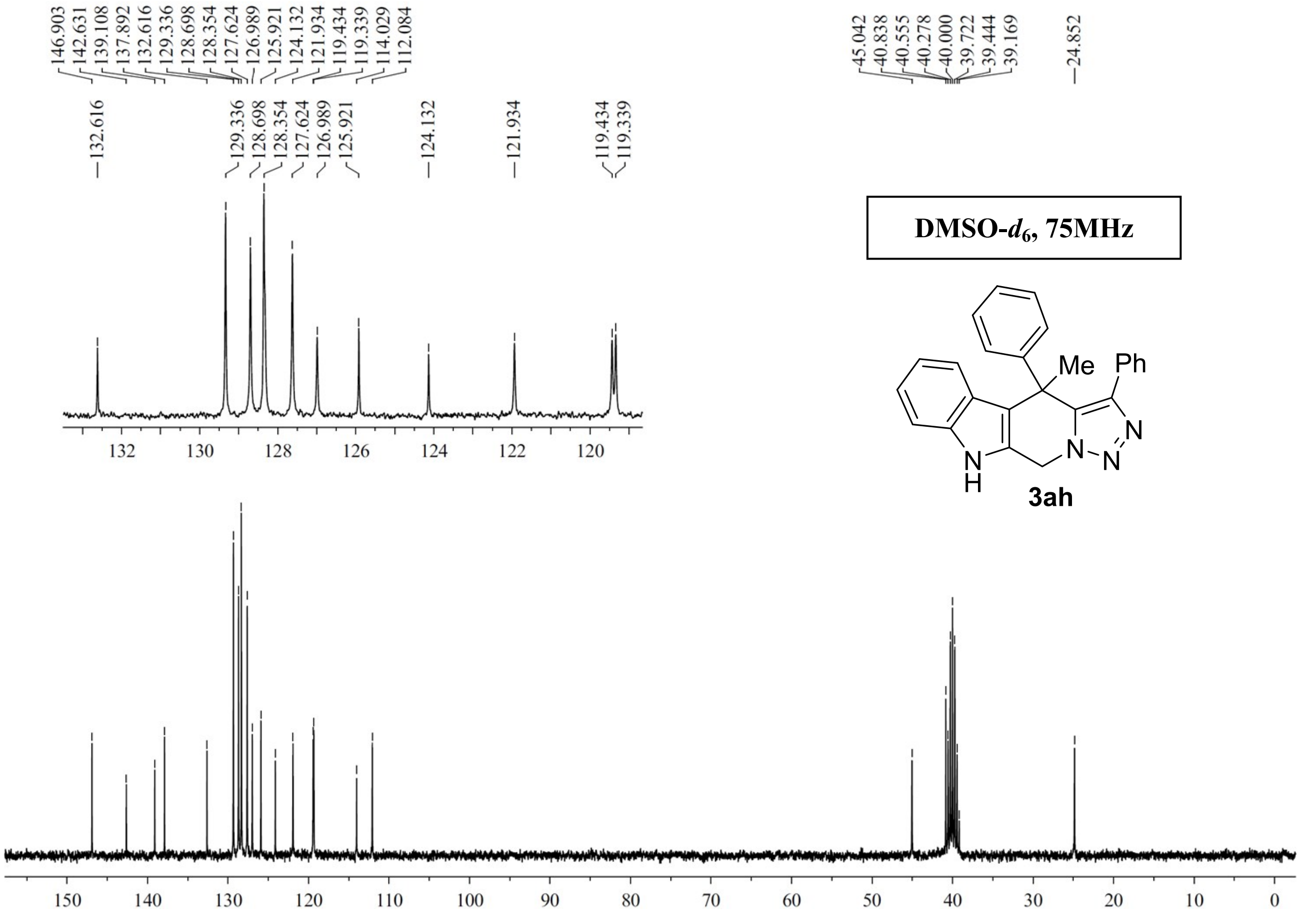
146.903 142.631 139.108 137.892 132.616 129.336 128.698 128.354 127.624 126.989 125.921 124.132 121.934 119.434 119.339 114.029 112.084
45.042 40.838 40.555 40.278 40.000 39.722 39.444 39.169
24.852
DMSO-d6, 75MHz
Me
Ph
N
H
3ah

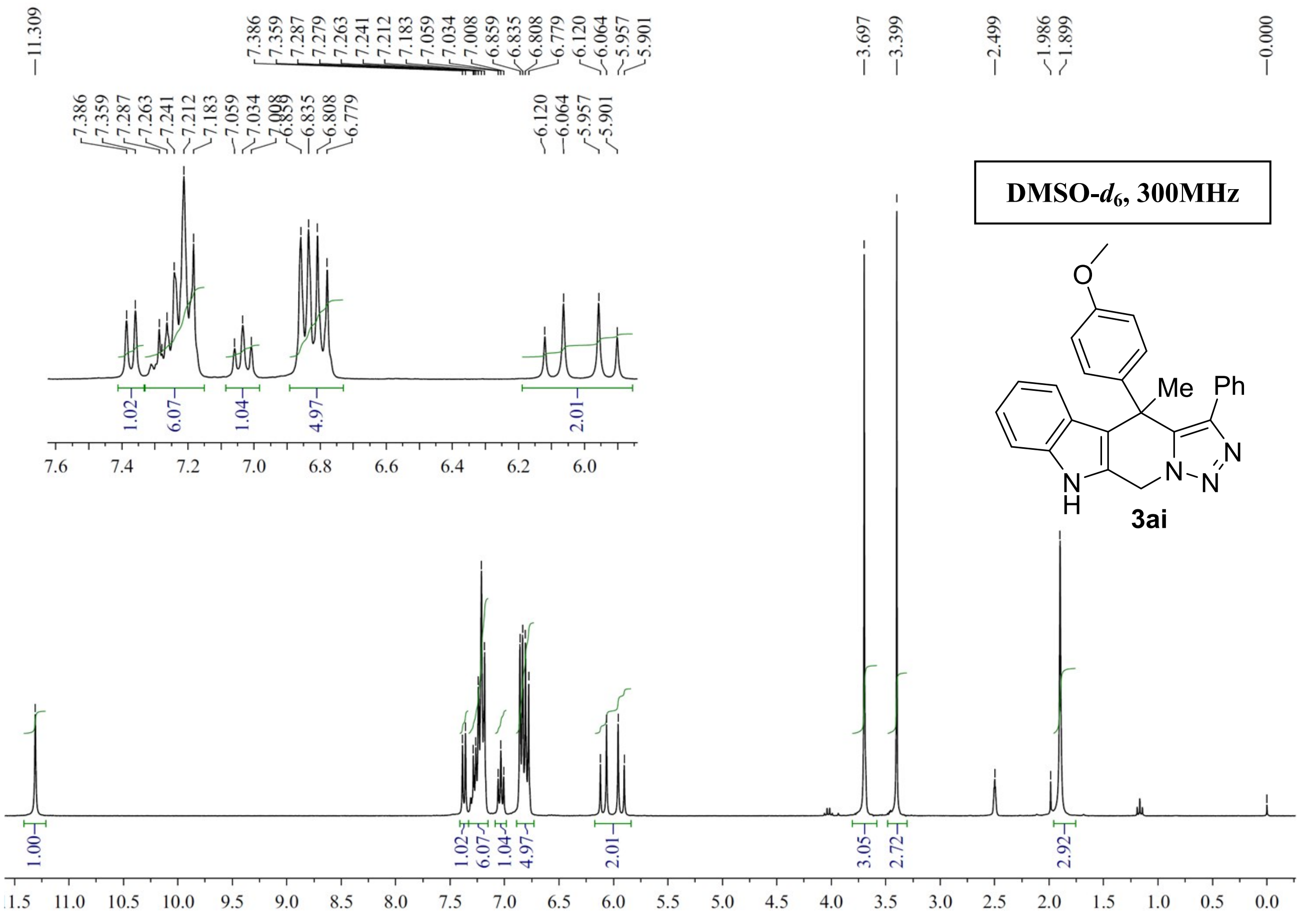
DMSO-d_6, 300MHz
Me
Ph
N
H
3ai

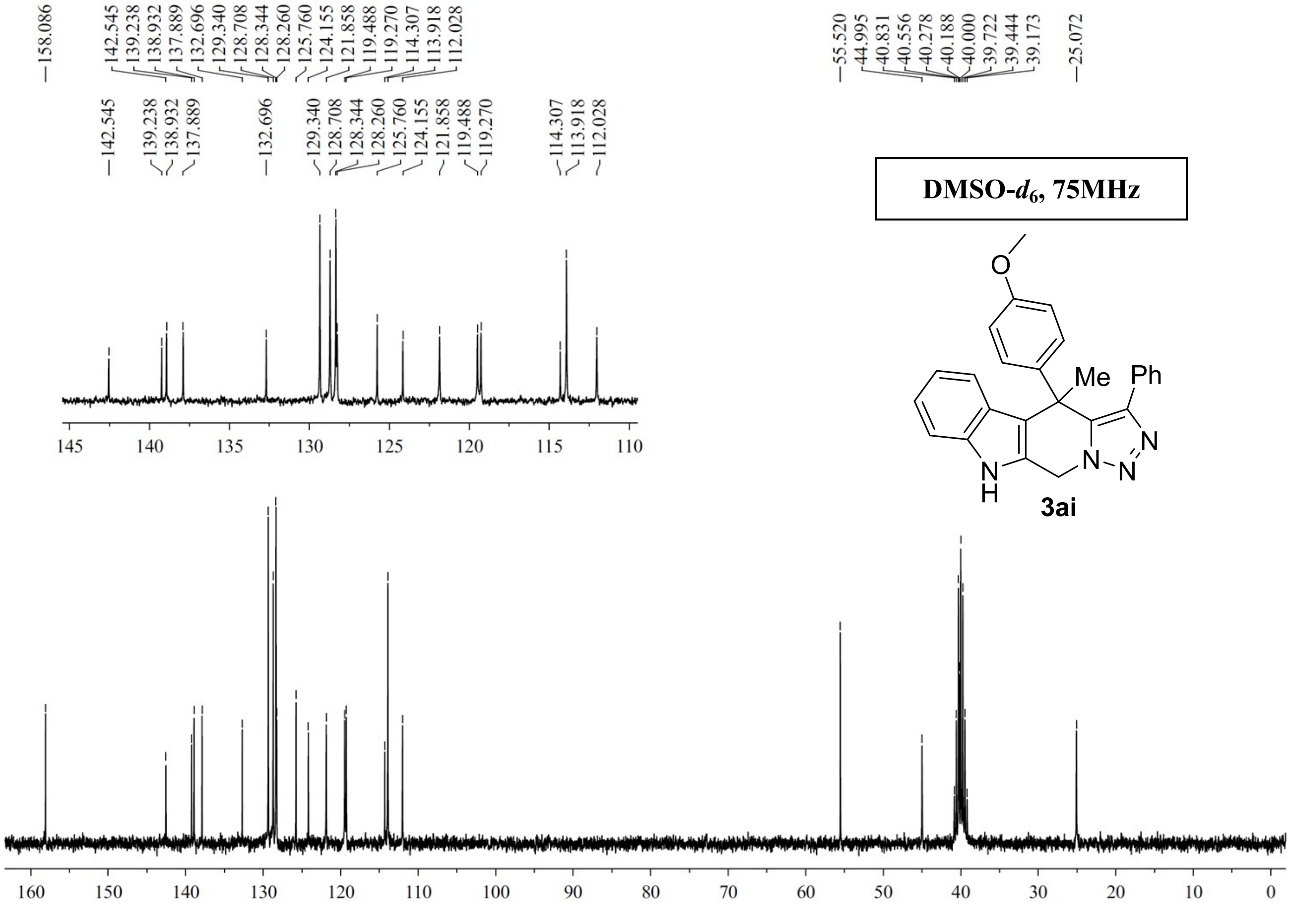
DMSO-d_6, 75MHz
Me
Ph
N
H
3ai

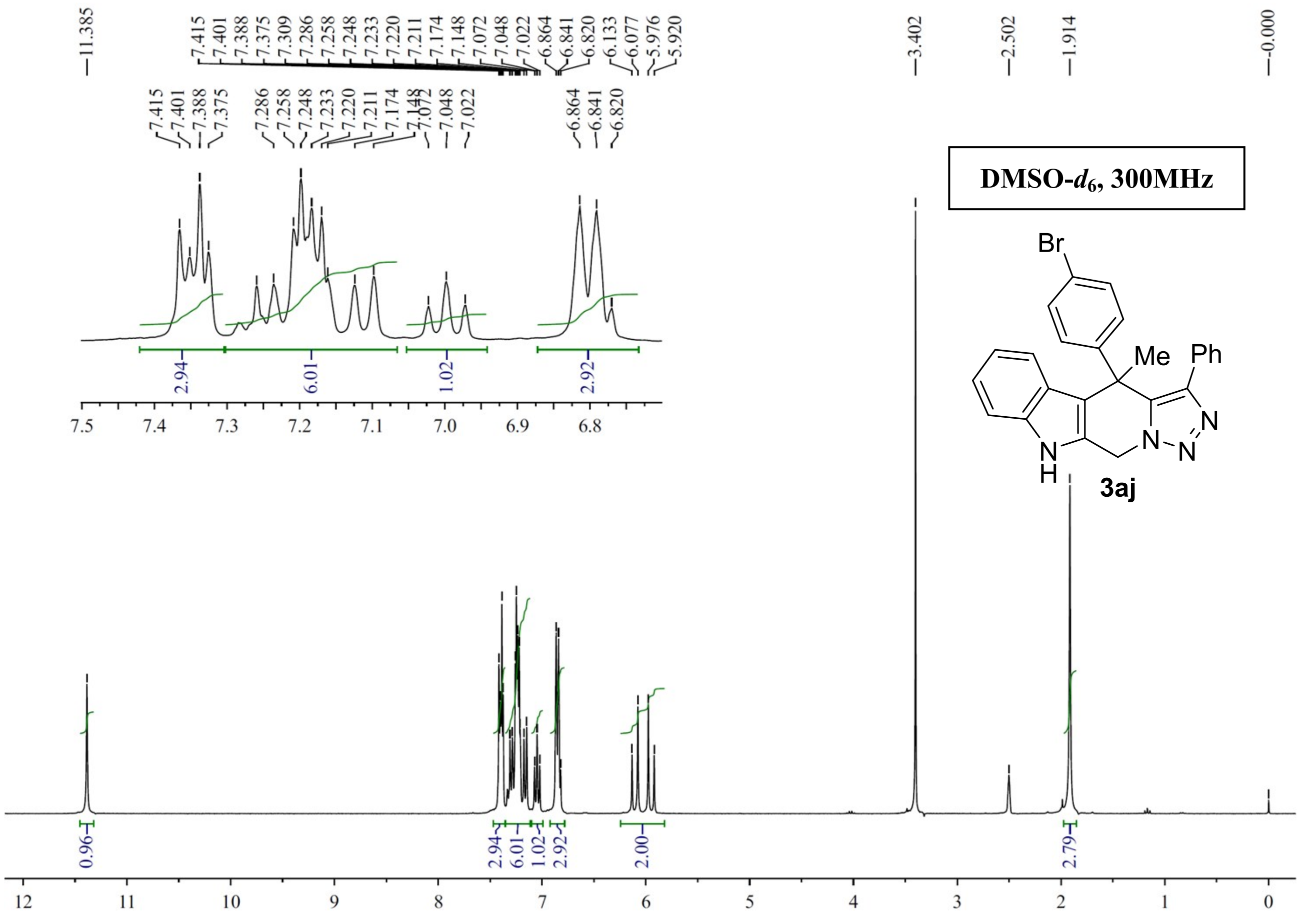
DMSO-d_6, 300MHz
Br
Me
Ph
N
H
3aj

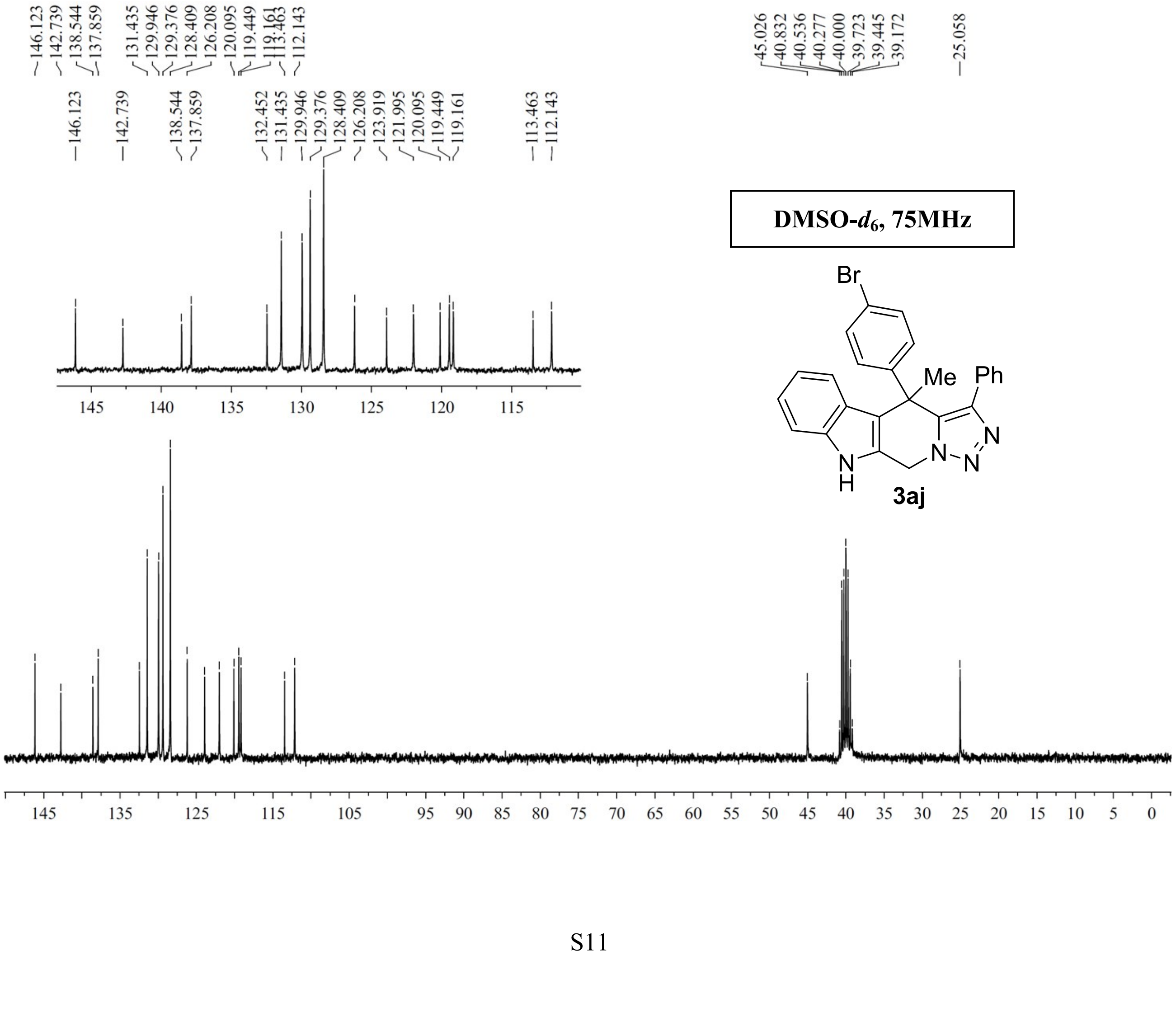
DMSO-d_6, 75MHz
Br
Me
Ph
N
H
3aj

DMSO-d_6, 300MHz

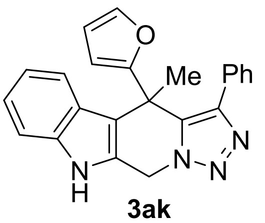

3ak

DMSO-d_6, 75MHz

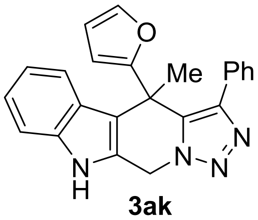

3ak

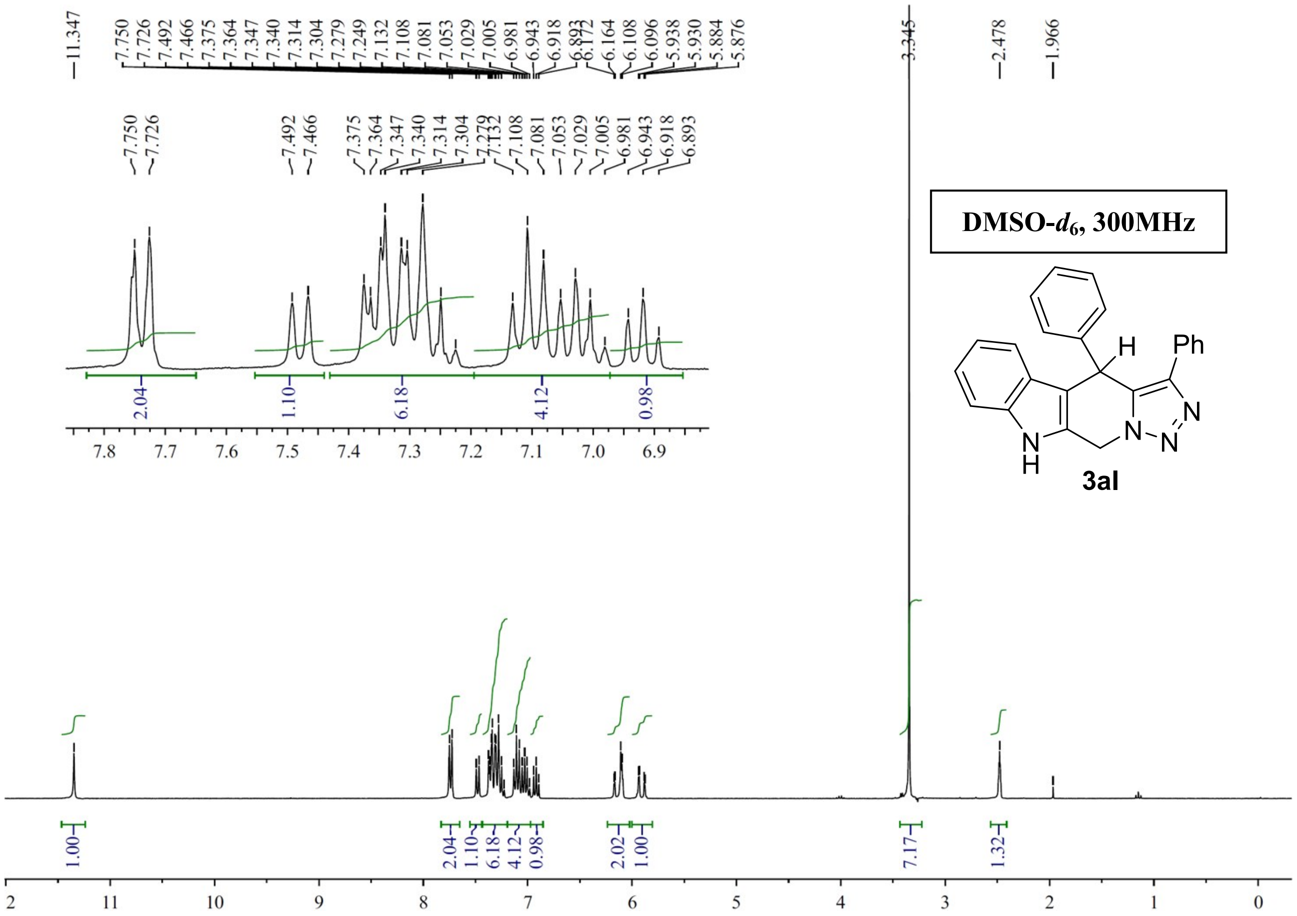
DMSO-d_6, 300MHz
3al

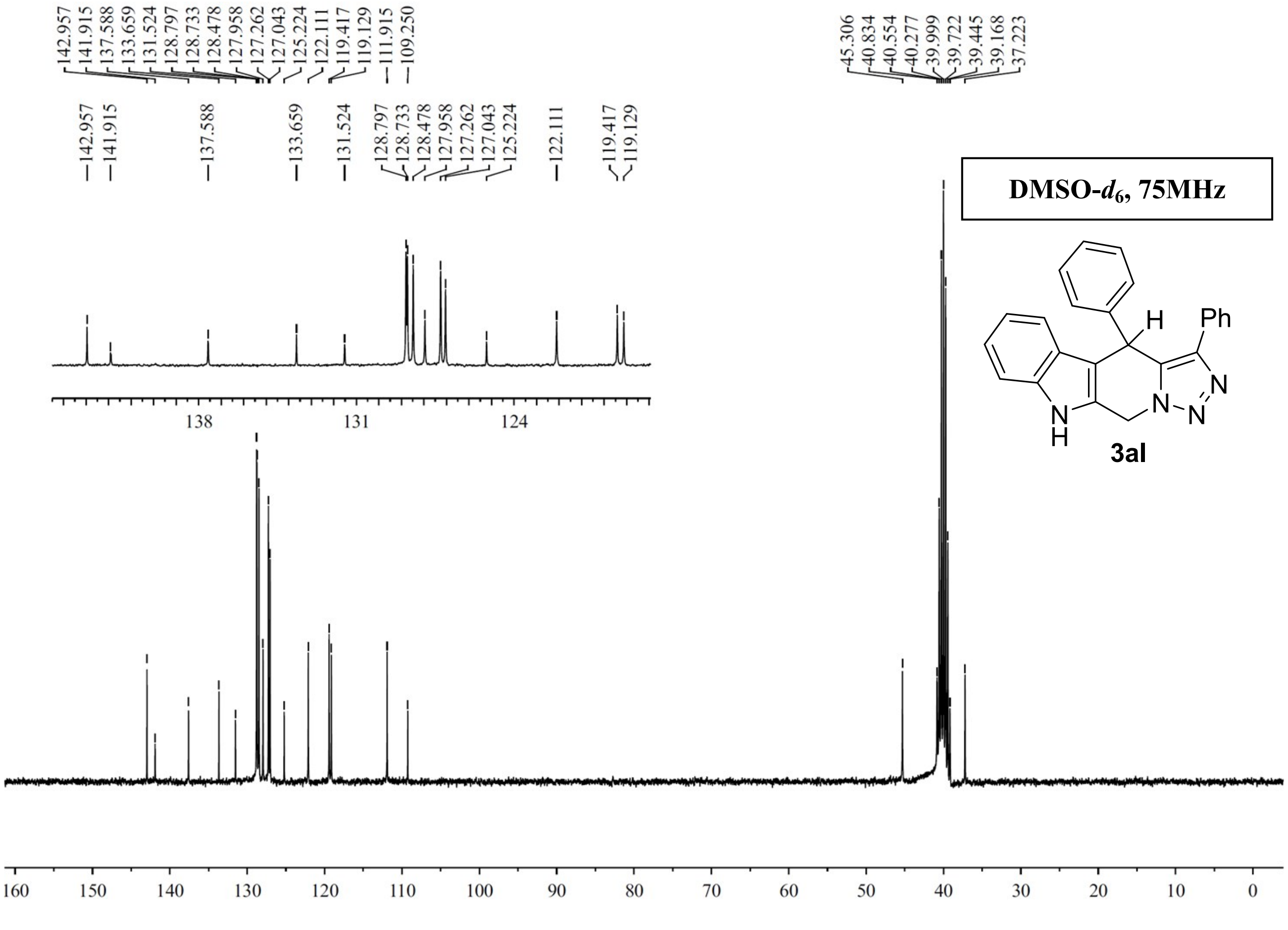
DMSO-d_6, 75MHz
3al

DMSO-d_6, 300MHz

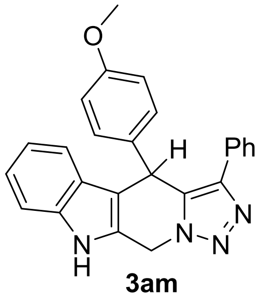

3am

DMSO-d_6, 75MHz

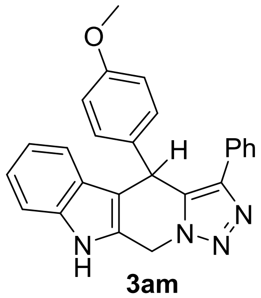

3am

DMSO-d_6, 300MHz

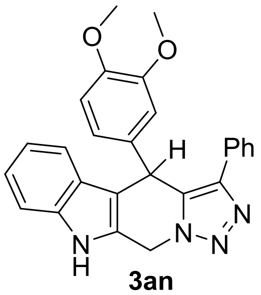

3an

DMSO-d_6, 75MHz

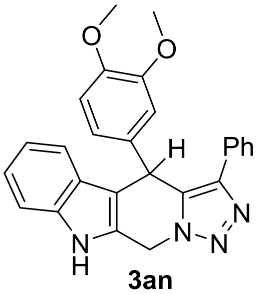

3an

DMSO-d_6, 300MHz

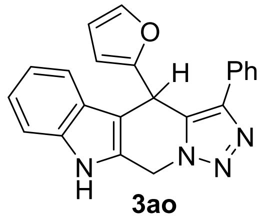

3ao

DMSO-d_6, 75MHz

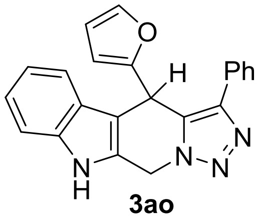

3ao

DMSO-d_6, 300MHz

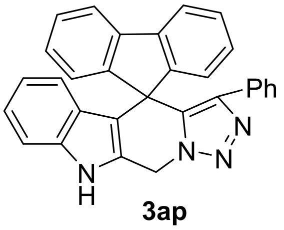

3ap

DMSO-d_6, 75MHz

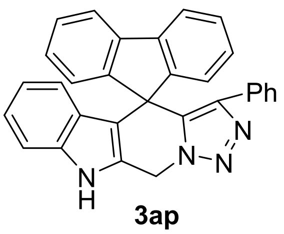

3ap

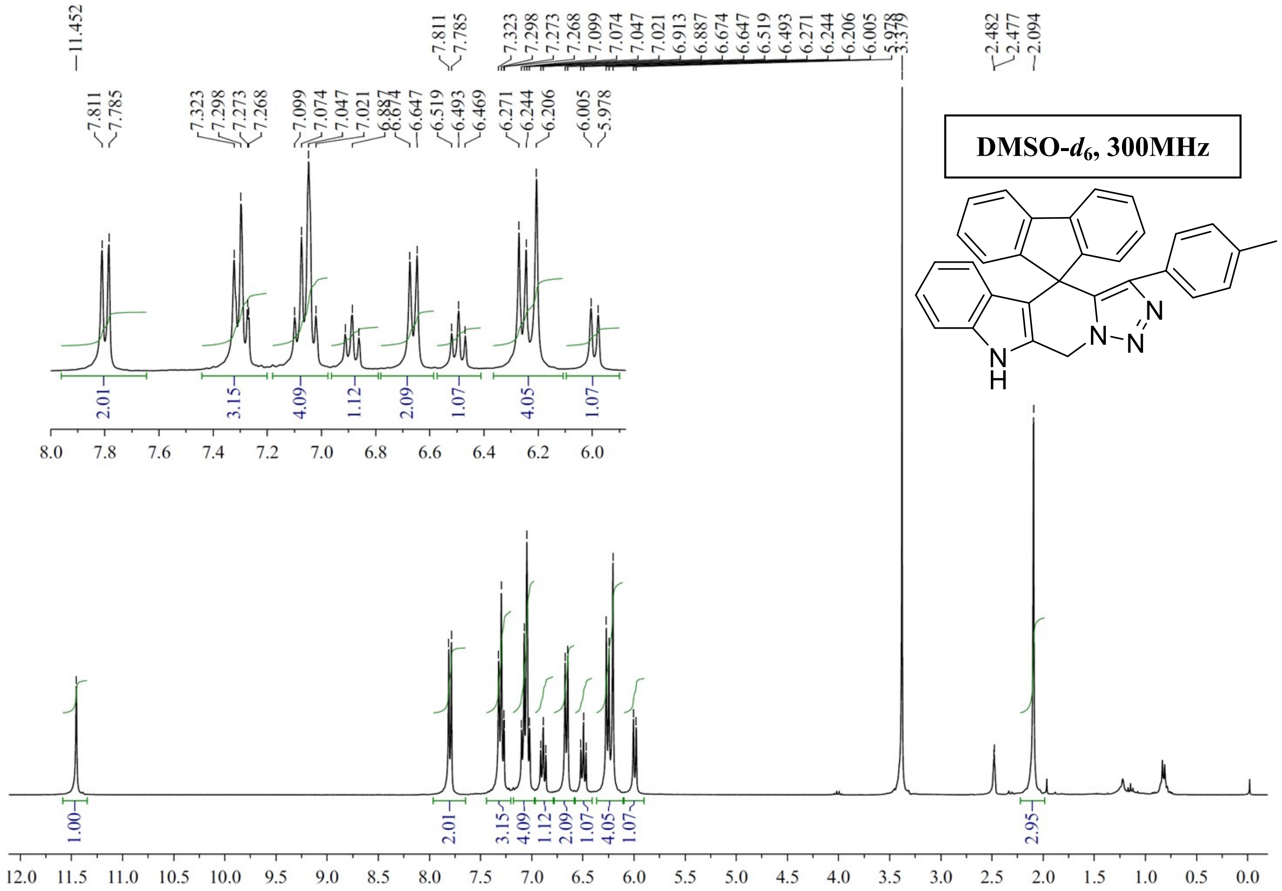
DMSO-d_6, 300MHz
11.452
7.811
7.785
7.323
7.298
7.273
7.268
7.099
7.074
7.047
7.021
6.913
6.887
6.674
6.647
6.519
6.493
6.469
6.271
6.244
6.206
6.005
5.978
3.379
2.482
2.477
2.094
2.01
3.15
4.09
1.12
2.09
1.07
4.05
1.07
1.00
2.95
N
N
N
H

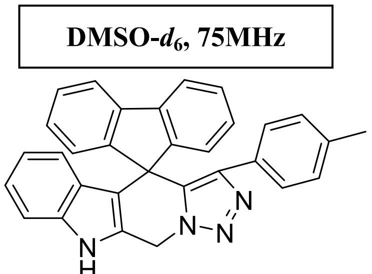
DMSO-d_6, 75MHz
N
N
N
H

3aq

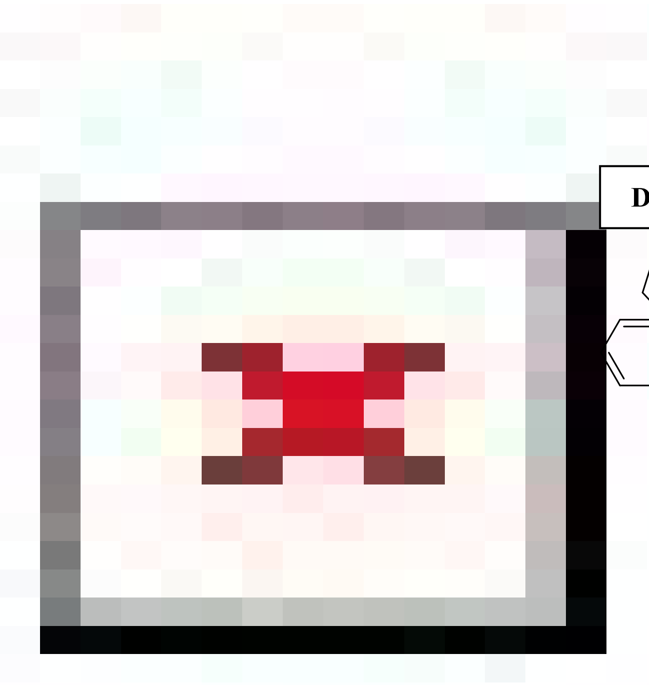

DMSO-d_6, 300MHz

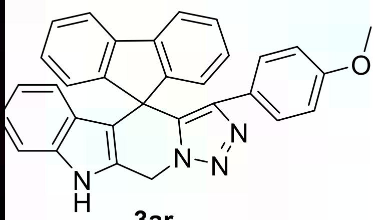

3ar

DMSO-d_6, 75MHz

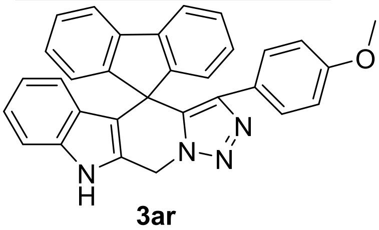

3ar

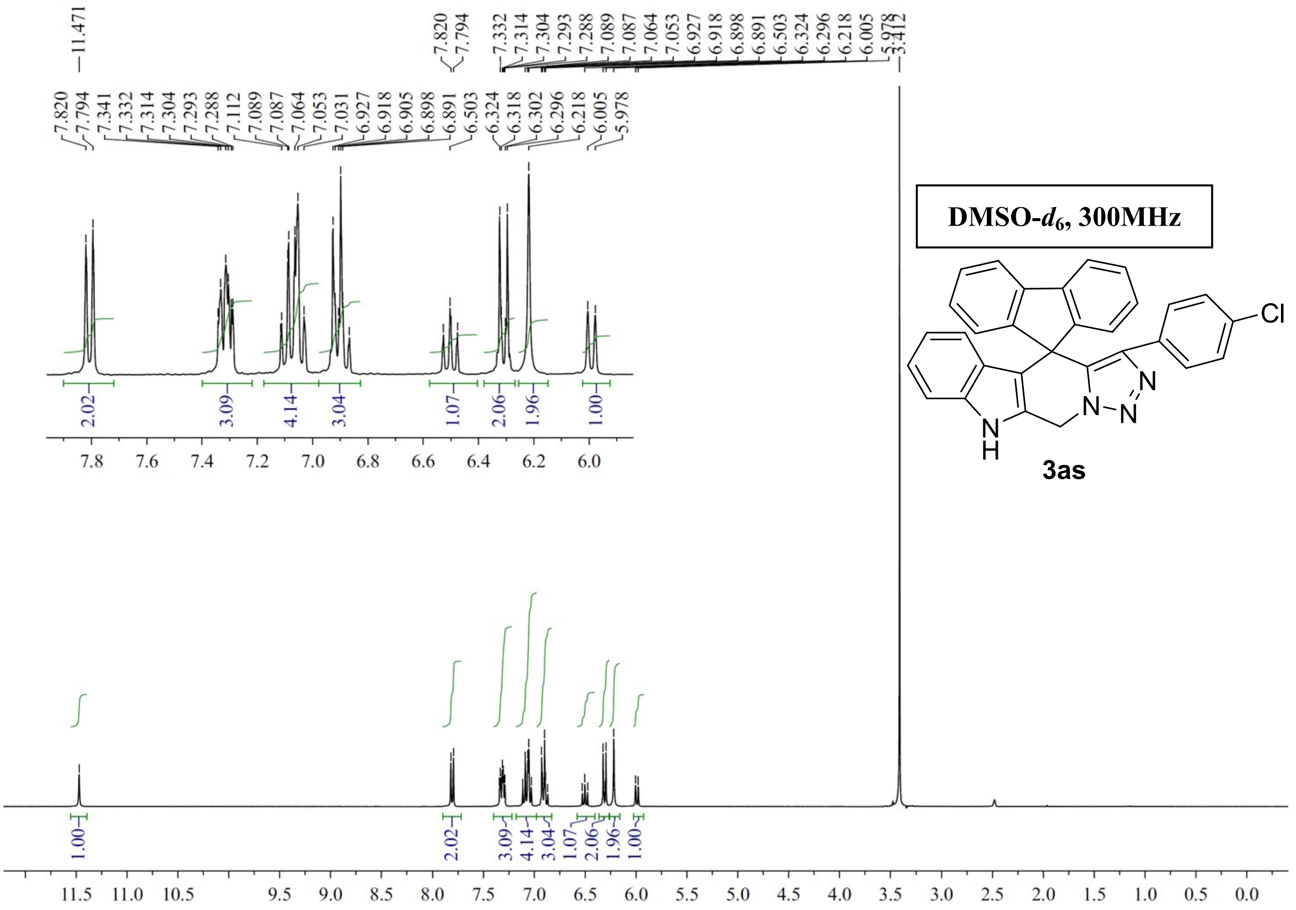

DMSO-d_6, 75MHz

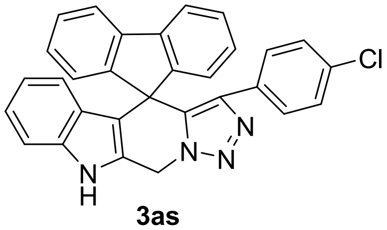

3as

DMSO-d_6, 300MHz

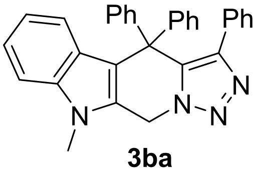

3ba

DMSO-d_6, 75MHz

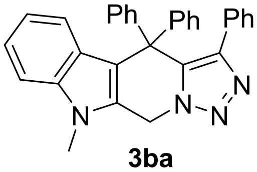

3ba

DMSO-d_6, 300MHz

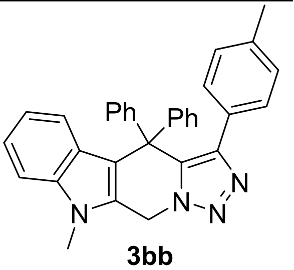

3bb

DMSO-d_6, 75MHz

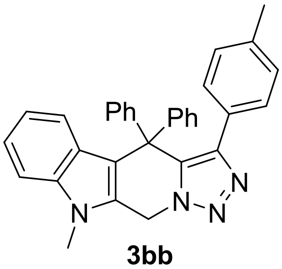

3bb

DMSO-d_6, 300MHz

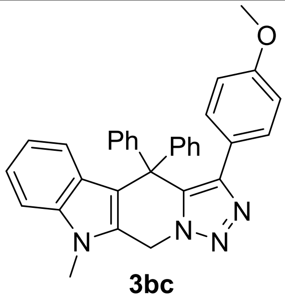

3bc

DMSO-d_6, 75MHz

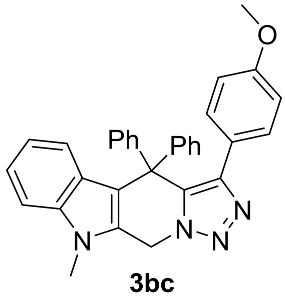

3bc

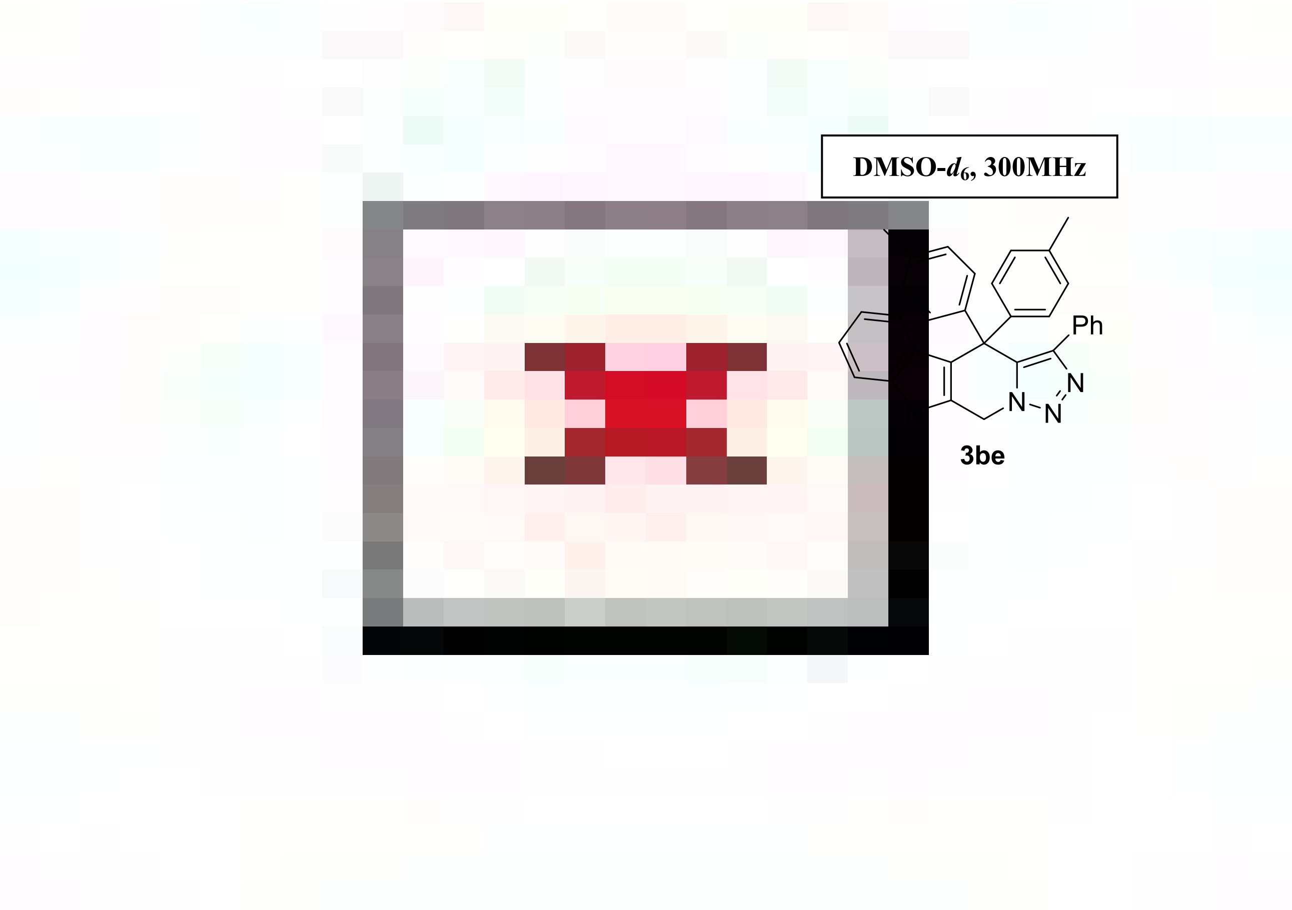
DMSO-d_6, 300MHz
Ph
N
N
N
3be

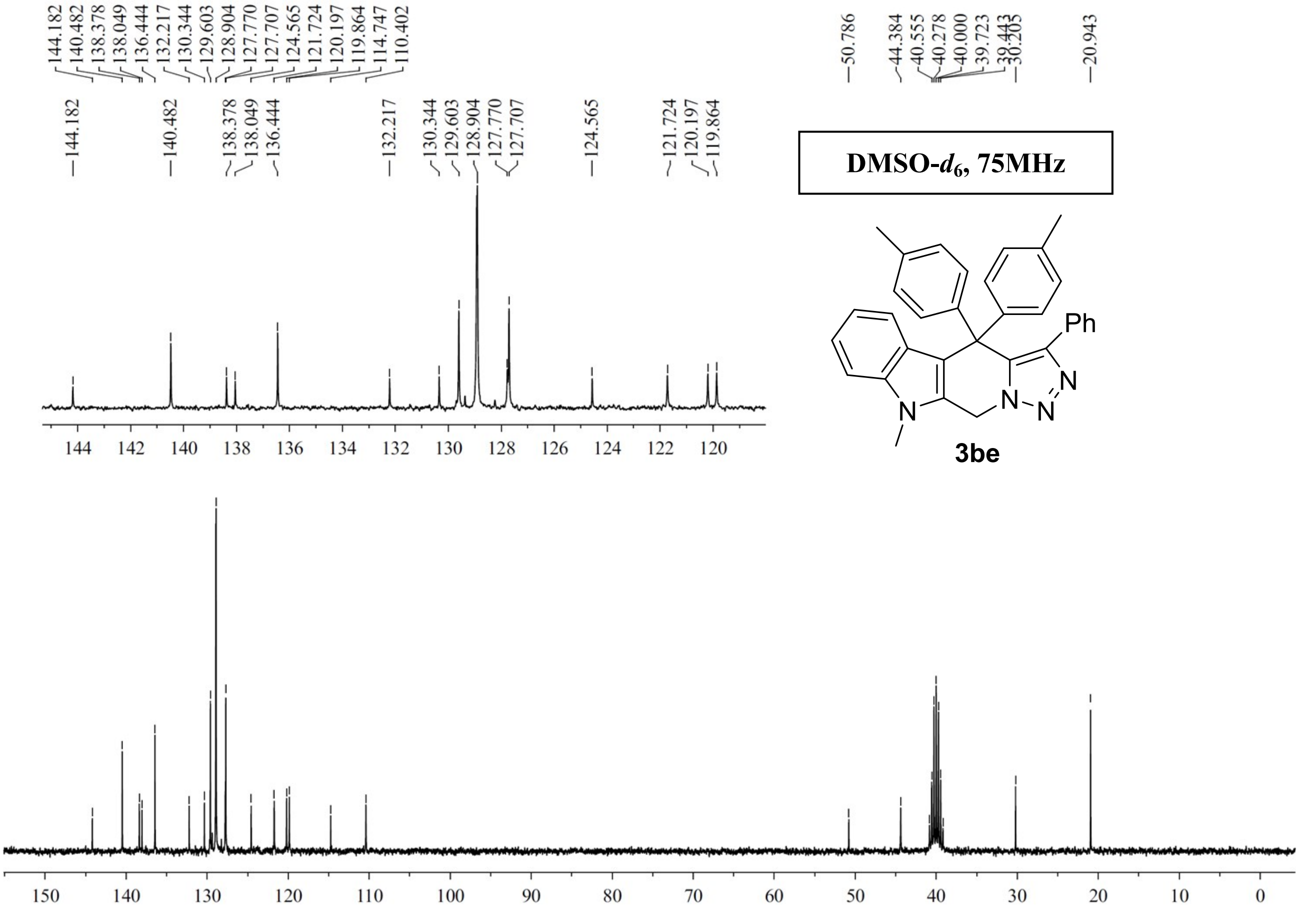
144.182
140.482
138.378
138.049
136.444
132.217
130.344
129.603
128.904
127.770
127.707
124.565
121.724
120.197
119.864
114.747
110.402
50.786
44.384
40.555
40.278
40.000
39.723
39.443
30.205
20.943
DMSO-d_6, 75MHz
Ph
N
N
N
N
3be
144 142 140 138 136 134 132 130 128 126 124 122 120
150 140 130 120 110 100 90 80 70 60 50 40 30 20 10 0

DMSO-d_6, 300MHz

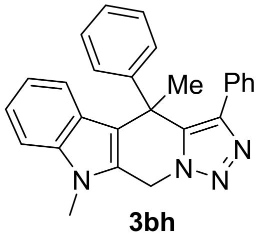

3bh

DMSO-d_6, 75MHz

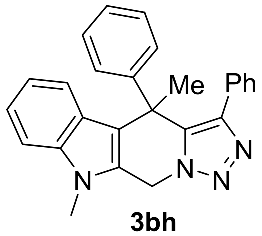

3bh

DMSO-d_6, 300MHz

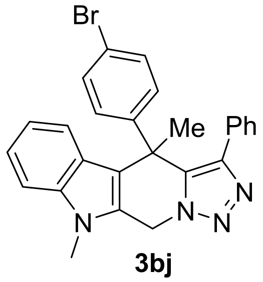

3bj

DMSO-d_6, 75MHz

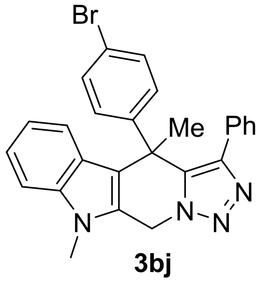

3bj

DMSO-d_6, 300MHz

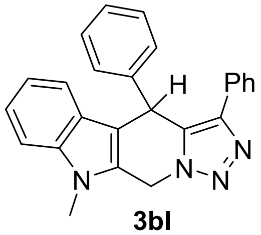

3bl

DMSO-d_6, 75MHz

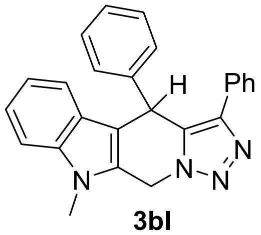

3bl

DMSO-d_6, 300MHz

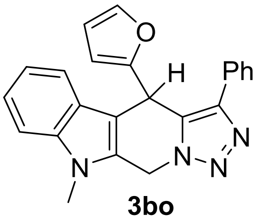

3bo

DMSO-d_6, 75MHz

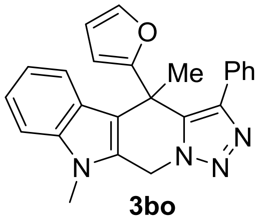

3bo

$CDCl_3$, 300MHz

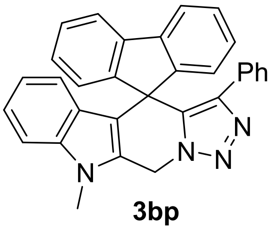

3bp

$CDCl_3$, 75MHz

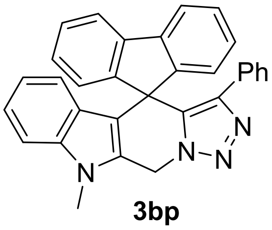

3bp

DMSO-d_6, 300MHz

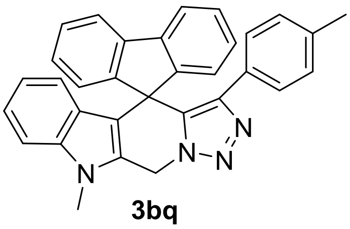

3bq

DMSO-d_6, 75MHz

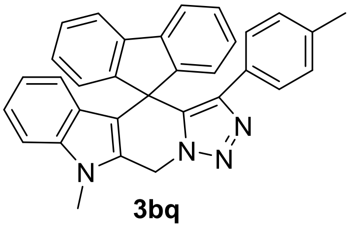

3bq

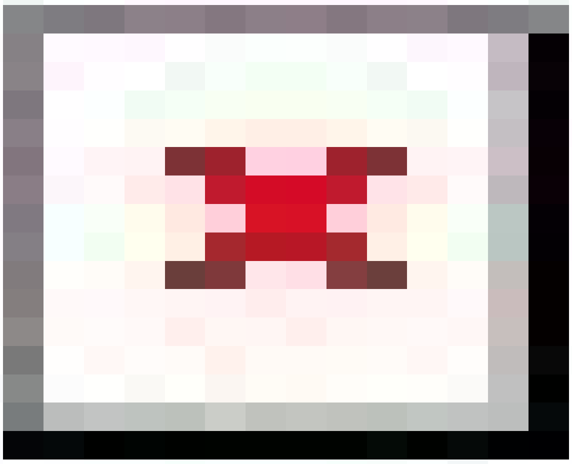

DMSO-d_6, 400MHz

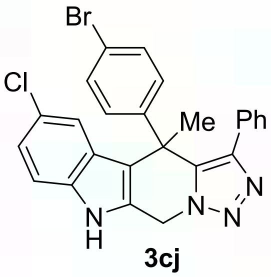

3cj

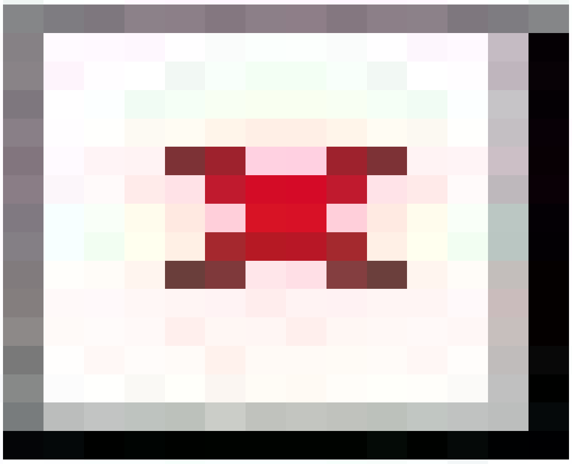

DMSO-d_6, 100MHz

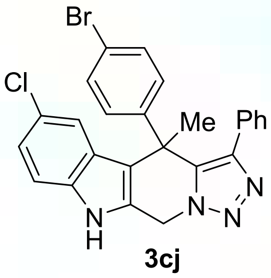

3cj

$CDCl_3$, 300MHz

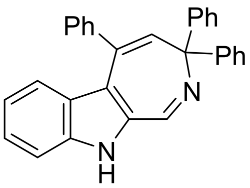

4aa

$CDCl_3$, 75MHz

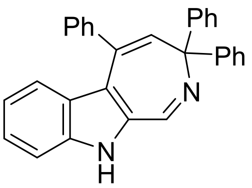

4aa

$CDCl_3$, 300MHz

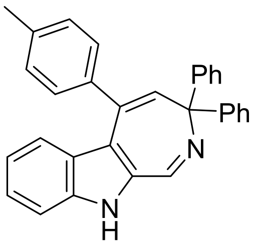

4ab

$CDCl_3$, 75MHz

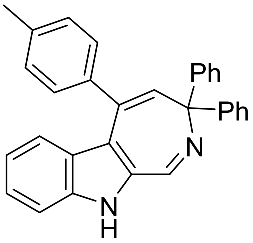

4ab

CDCl$_3$, 300MHz

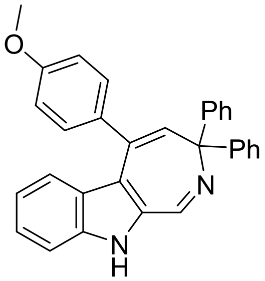

4ac

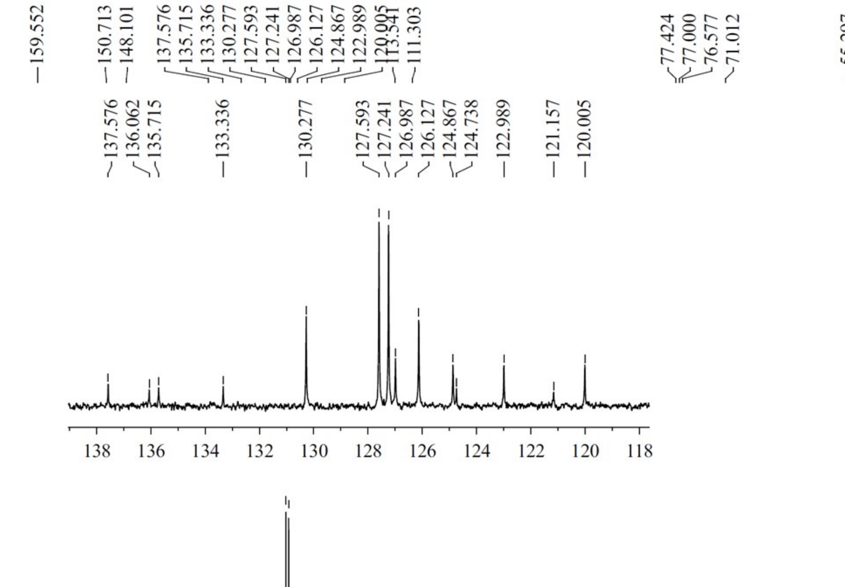

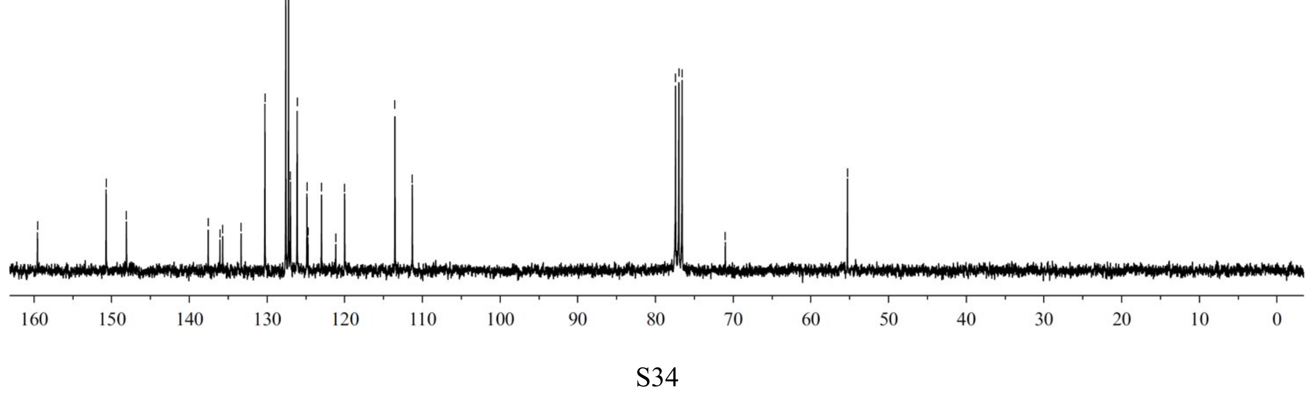

CDCl$_3$, 75MHz

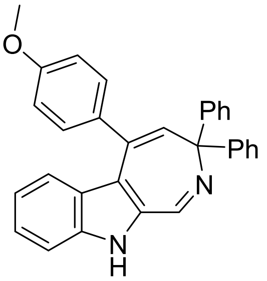

4ac

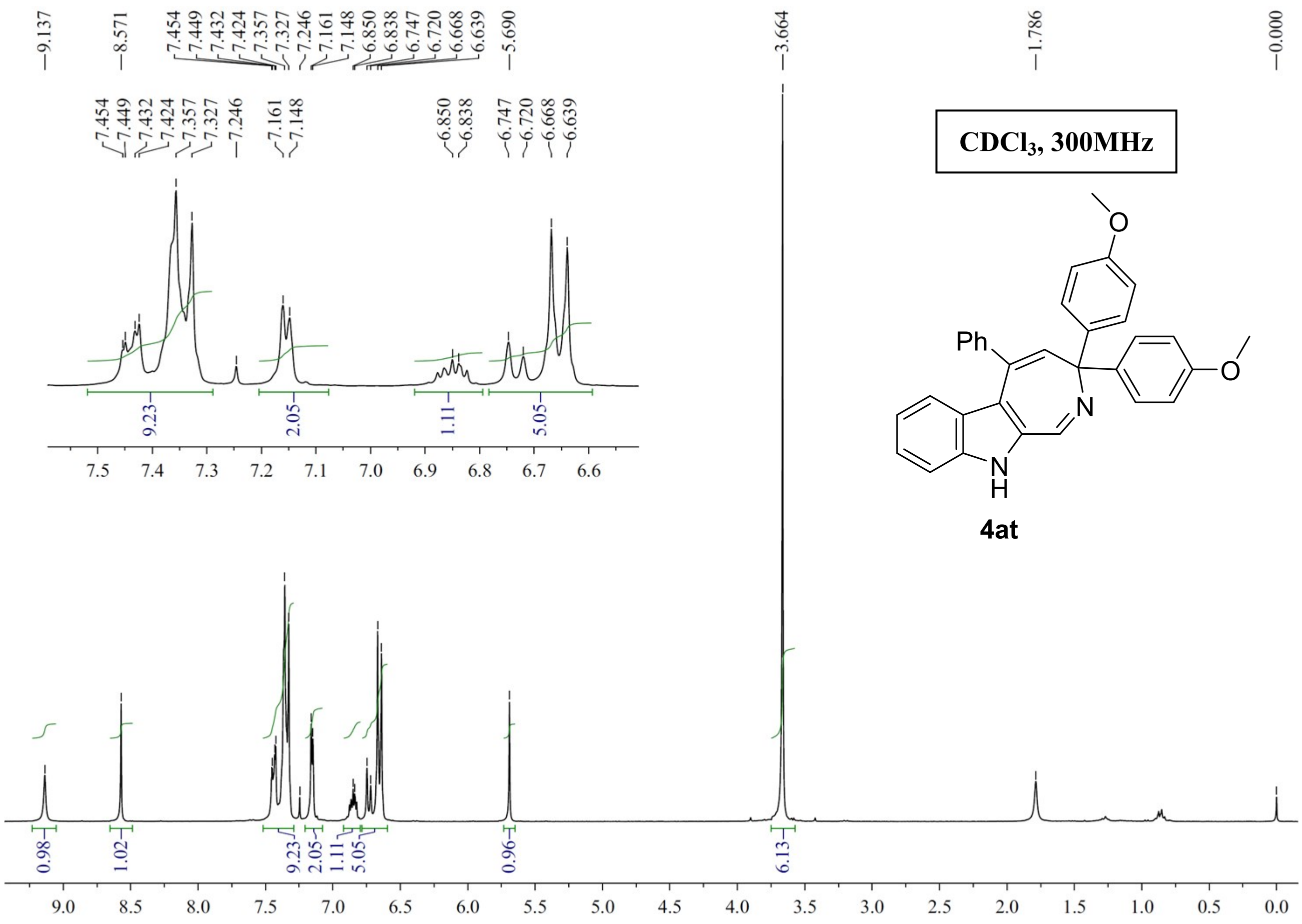
9.137
8.571
7.454
7.449
7.432
7.424
7.357
7.327
7.246
7.161
7.148
6.850
6.838
6.747
6.720
6.668
6.639
5.690
3.664
1.786
0.000
CDCl3, 300MHz
Ph
N
H
O
4at
9.23
2.05
1.11
5.05
0.98
1.02
0.96
6.13

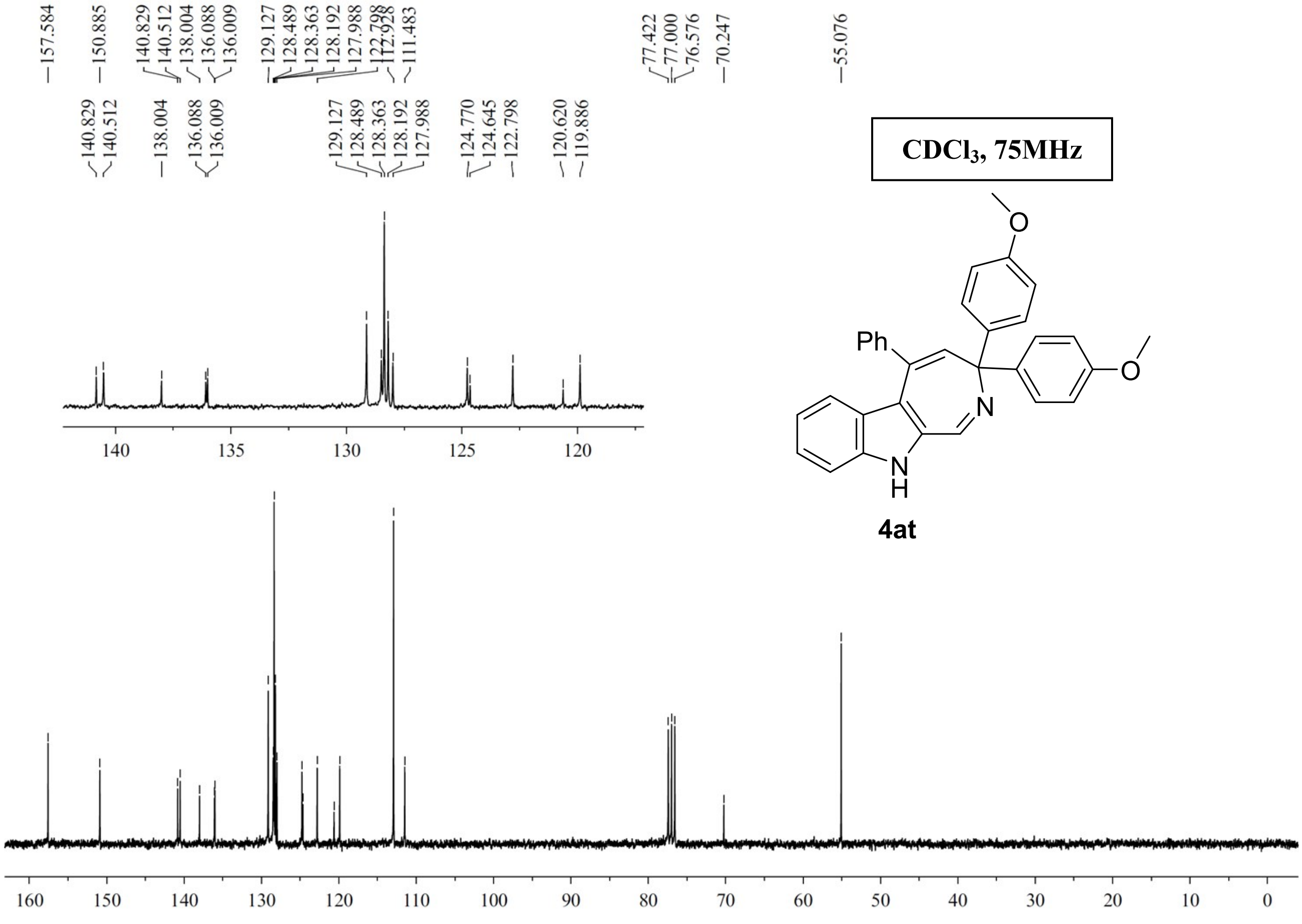
157.584
150.885
140.829
140.512
138.004
136.088
136.009
129.127
128.489
128.363
128.192
127.988
122.798
112.928
111.483
124.770
124.645
120.620
119.886
77.422
77.000
76.576
70.247
55.076
CDCl3, 75MHz
Ph
N
H
O
4at

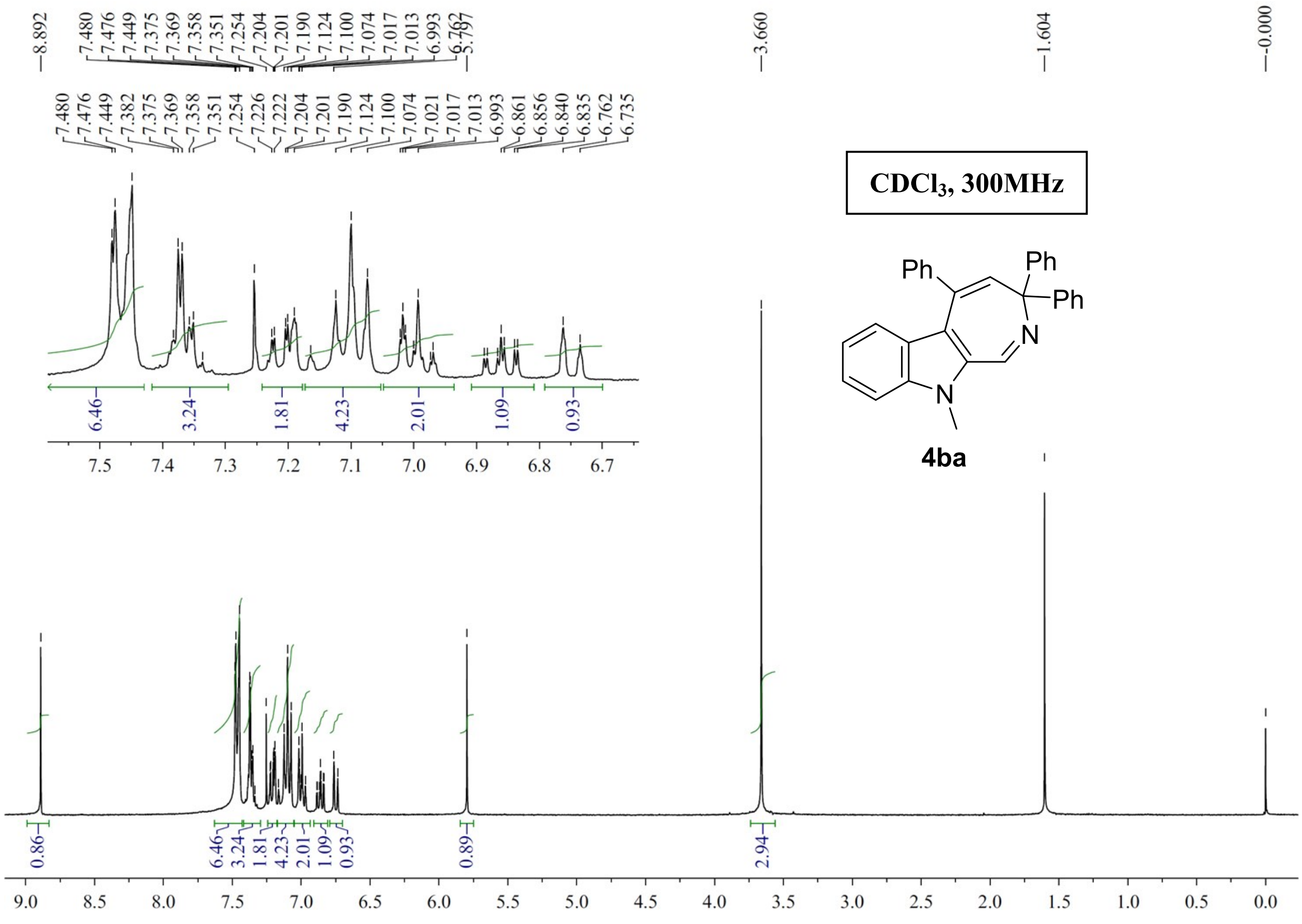

$CDCl_3$, 300MHz

4ba

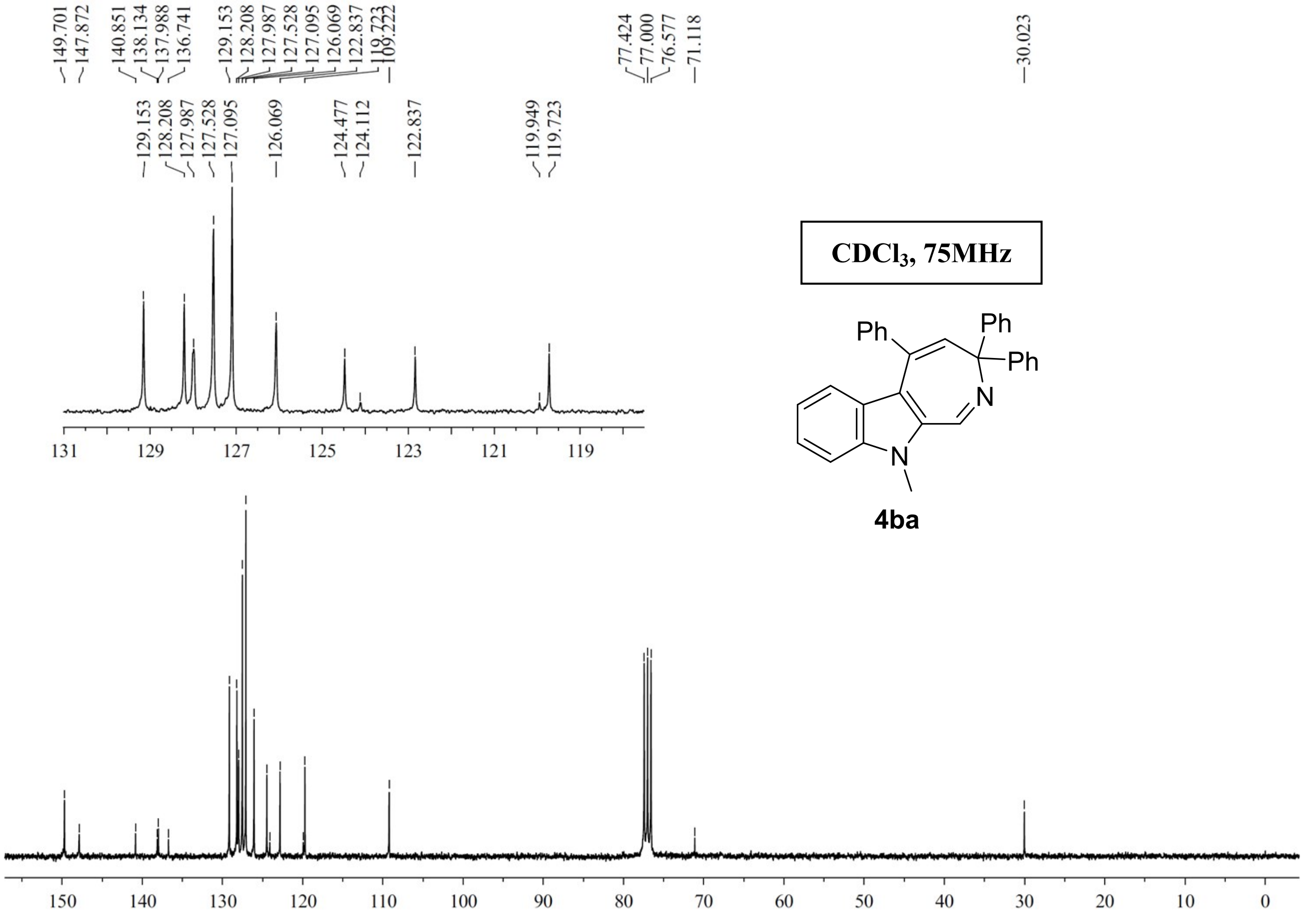

$CDCl_3$, 75MHz

4ba

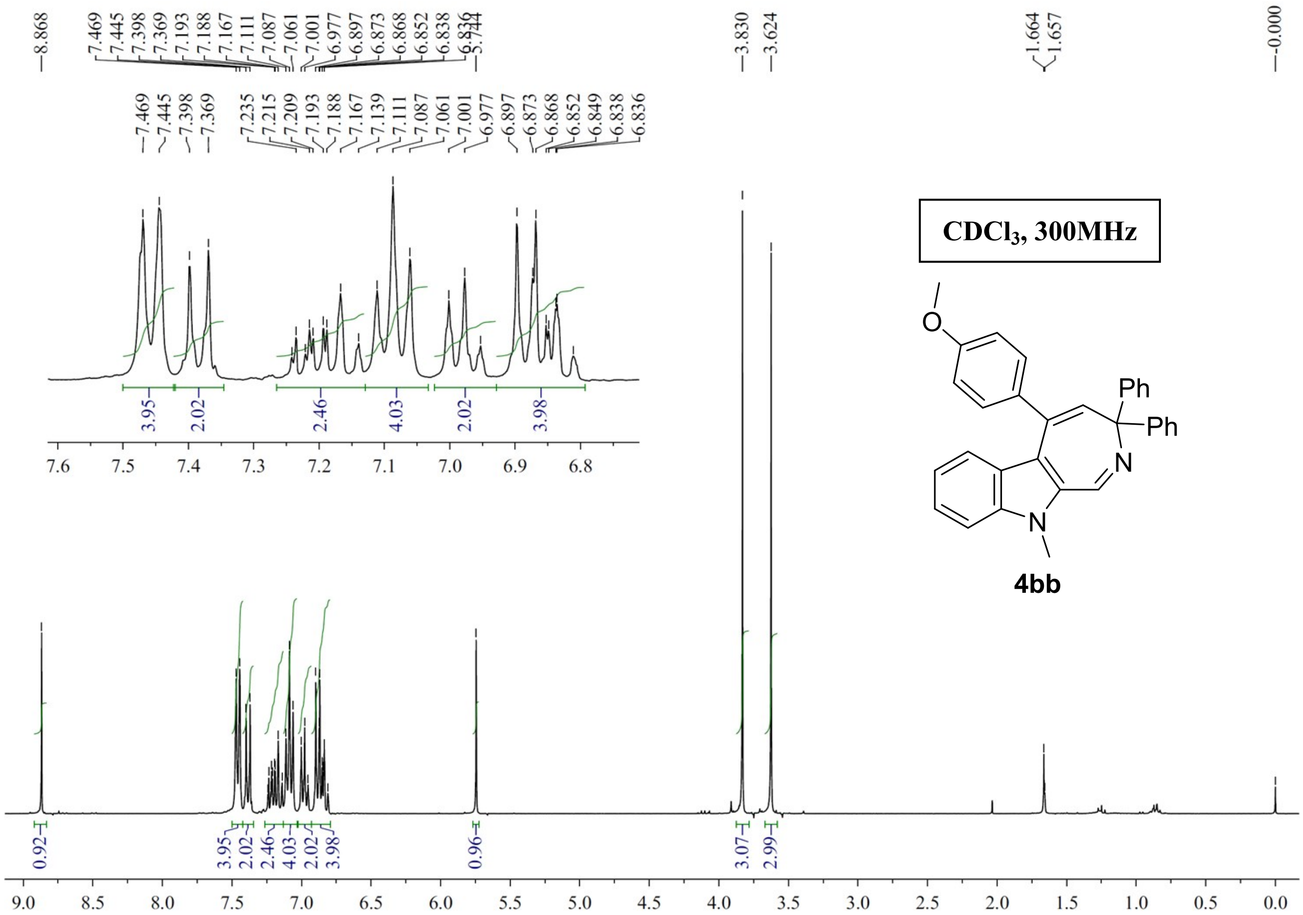
CDCl3, 300MHz
O
Ph
Ph
N
N
4bb

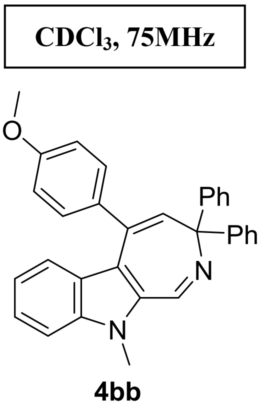
CDCl3, 75MHz
O
Ph
Ph
N
N
4bb

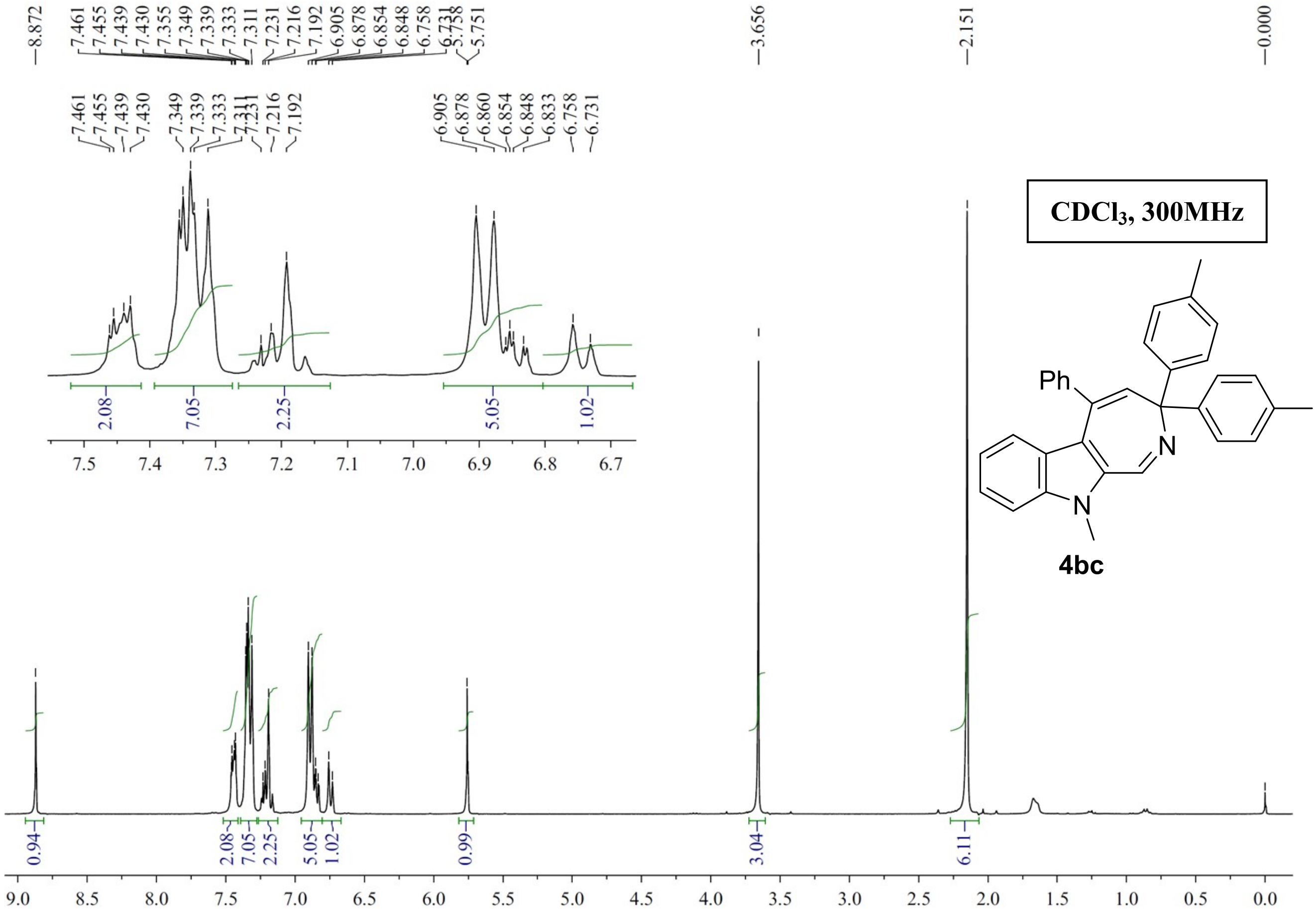
CDCl3, 300MHz
Ph
N
N
4bc
0.94
2.08
7.05
2.25
5.05
1.02
0.99
3.04
6.11

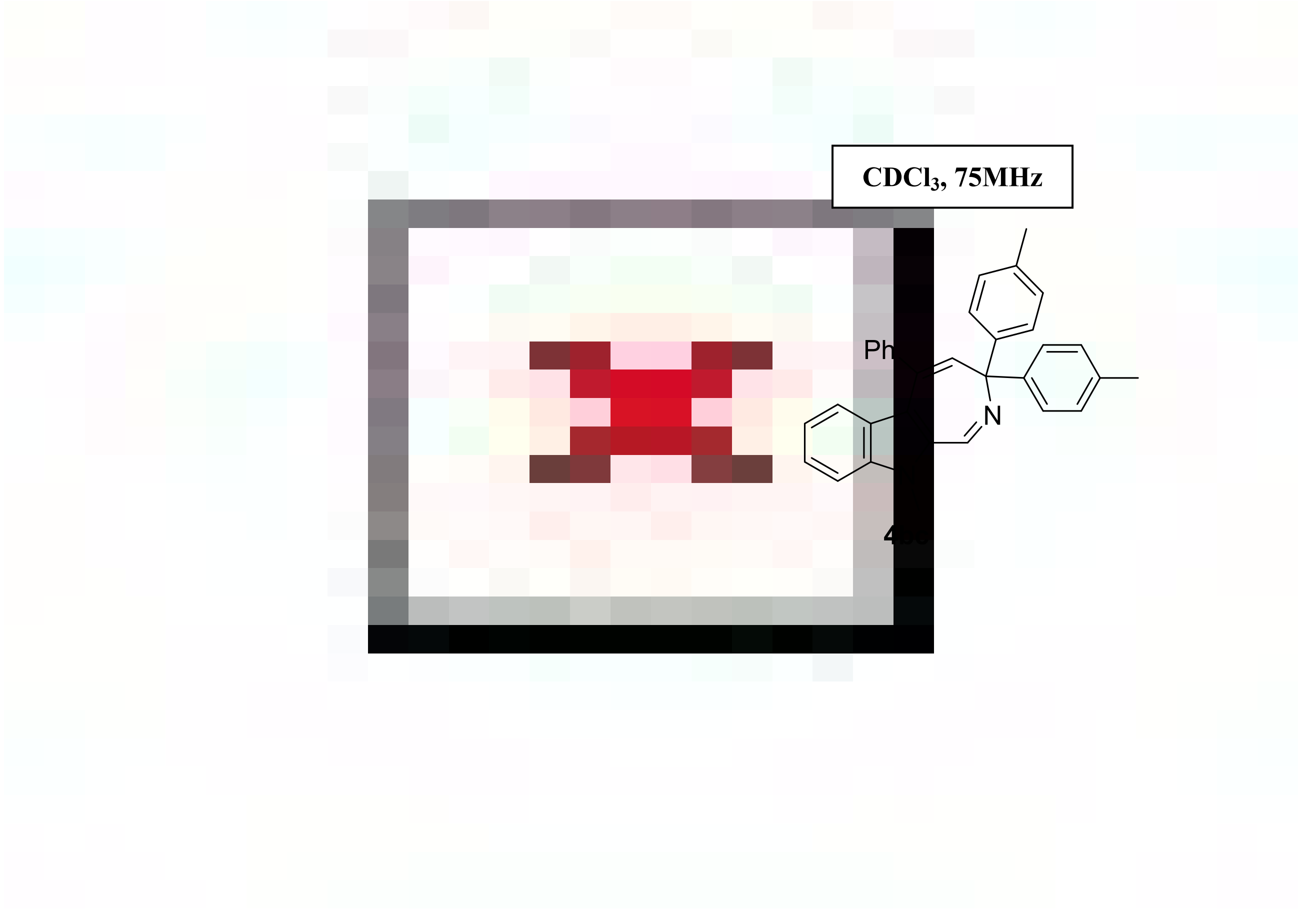
CDCl3, 75MHz
Ph
N

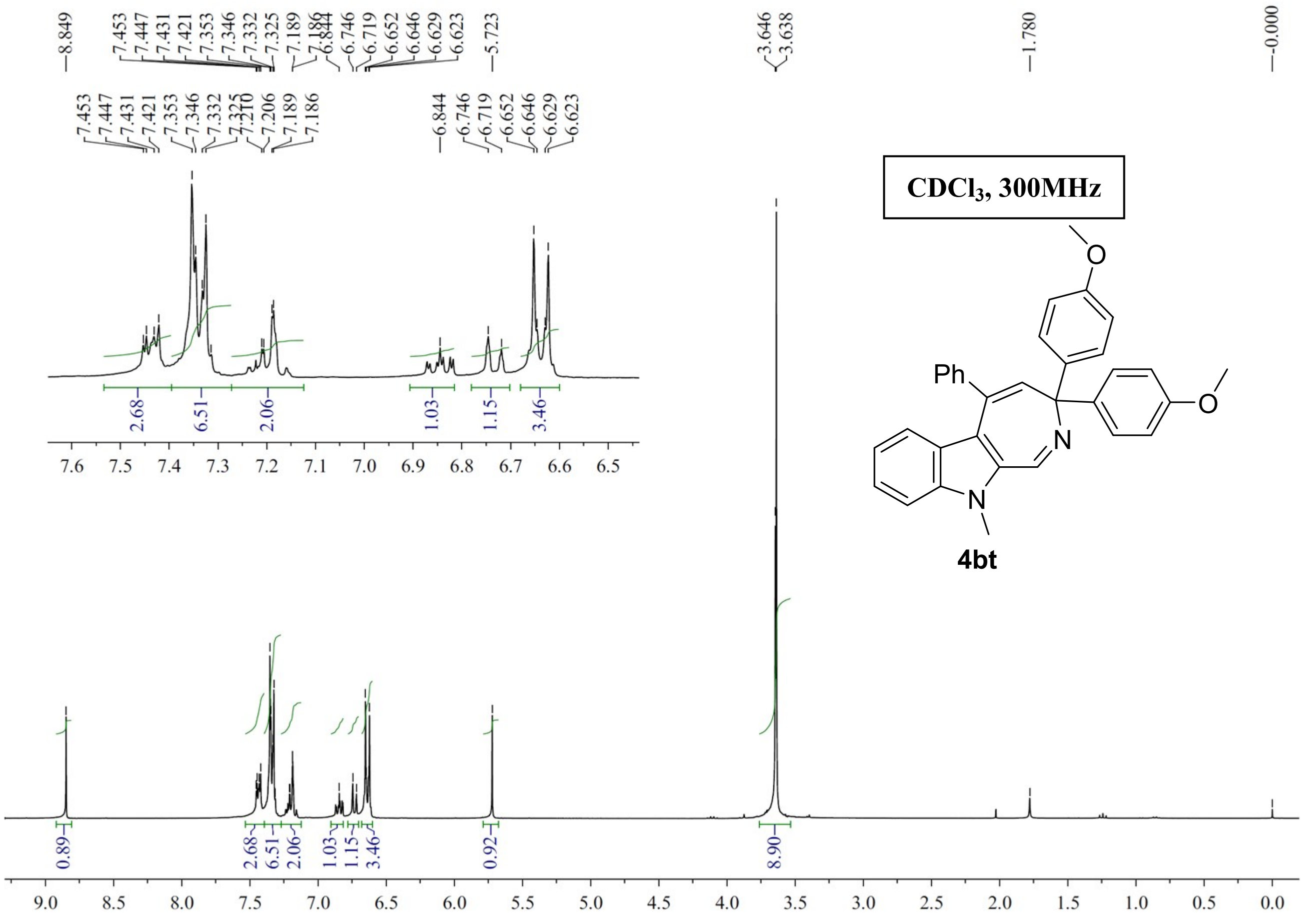
CDCl$_3$, 300MHz
Ph
N
O
4bt

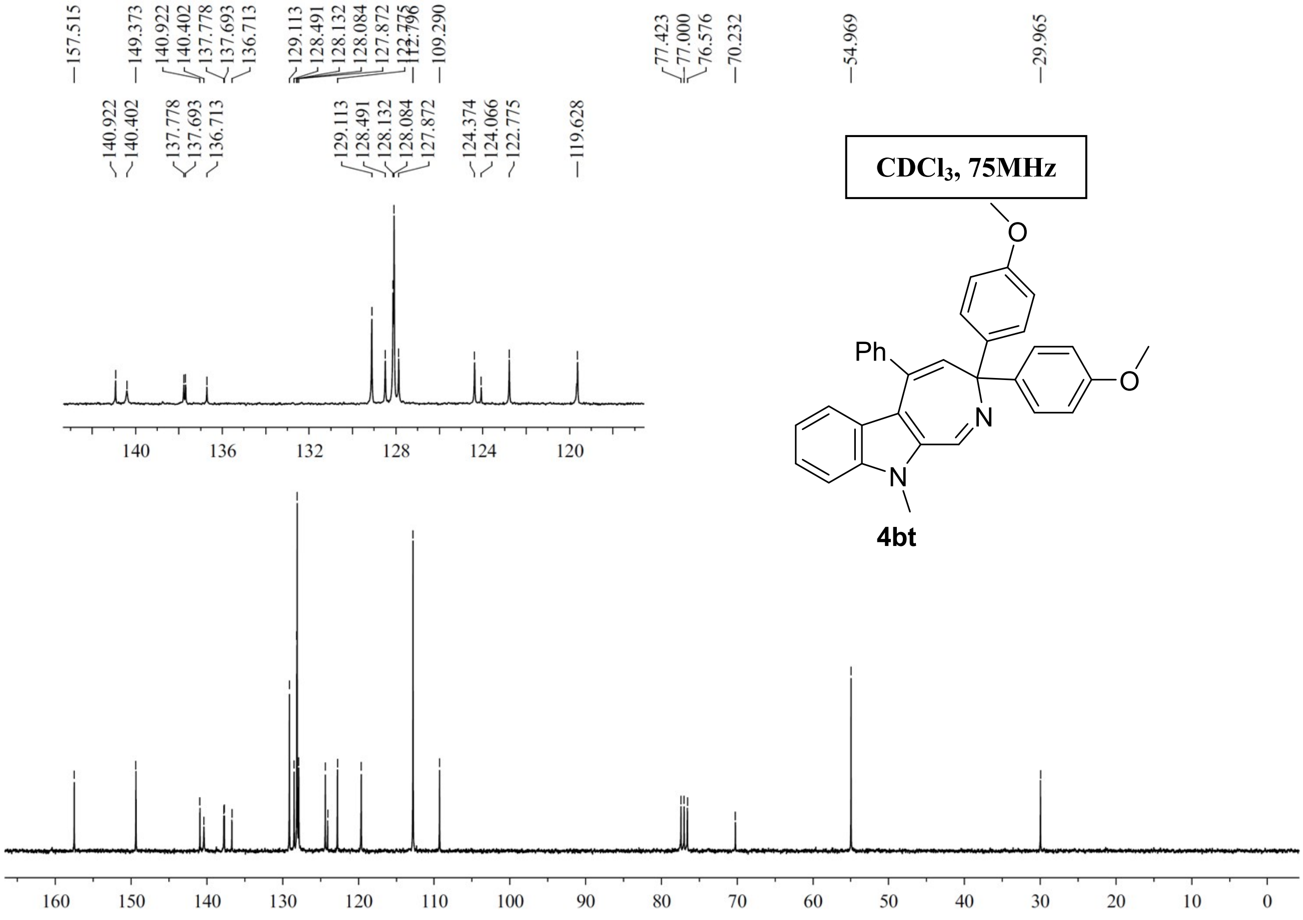
CDCl$_3$, 75MHz
Ph
N
O
4bt
